# Development and Application of a Prophage Integrase Typing Scheme for Group B *Streptococcus*

**DOI:** 10.3389/fmicb.2020.01993

**Published:** 2020-08-31

**Authors:** Chiara Crestani, Taya L. Forde, Ruth N. Zadoks

**Affiliations:** ^1^Institute of Biodiversity, Animal Health and Comparative Medicine, University of Glasgow, Glasgow, United Kingdom; ^2^Sydney School of Veterinary Science, University of Sydney, Camden, NSW, Australia

**Keywords:** prophage, bacteriophage, group B *Streptococcus*, *Streptococcus agalactiae*, integrase, phage typing, phage-inducible chromosomal island, PICI

## Abstract

Group B *Streptococcus* (GBS) is a gram-positive pathogen mainly affecting humans, cattle, and fishes. Mobile genetic elements play an important role in the evolution of GBS, its adaptation to host species and niches, and its pathogenicity. In particular, lysogenic prophages have been associated with a high virulence of certain strains and with their ability to cause invasive infections in humans. It is therefore important to be able to accurately detect and classify prophages in GBS genomes. Several bioinformatic tools for the identification of prophages in bacterial genomes are available on-line. However, genome searches for most of these programs are affected by the composition of their reference database. Lack of databases specific to GBS results in failure to recognize all prophages in the species. Additionally, performance of these programs is affected by genome fragmentation in the case of draft genomes, leading to underestimation of the number of phages. They also prove impractical when dealing with large genome datasets and they do not offer a quick way of classifying bacteriophages. We developed a GBS-specific method to screen genome assemblies for the presence of prophages and to classify them based on a reproducible typing scheme. This was achieved through an extensive search of a vast number of high-quality GBS sequences (*n* = 572) originating from different host species and countries in order to build a database of phage integrase types, on which the scheme is based. The proposed typing scheme comprises 12 integration sites and sixteen prophage integrase types, including multiple subtypes per integration site and integrase genes that were not site-specific. Two putative phage-inducible chromosomal islands (PICI) and their insertion sites were also identified during the course of these analyses. Phages were common and diverse in all major clonal complexes associated with human disease and detected in isolates from every animal species and continent included in the study. This database will facilitate further work on the prevalence and role of prophages in GBS evolution, and identifies the roles of PICIs in GBS and of prophage in hypervirulent ST283 as areas for further research.

## 1. Introduction

Group B *Streptococcus* (GBS)—also known as *Streptococcus agalactiae*—is a gram-positive bacterium with a wide host range (Brochet et al., 2006; Delannoy et al., 2016; Richards et al., 2019). Major hosts of interest from a public health and socio-economic point of view are humans (High et al., 2005; Le Doare et al., 2017; Seale et al., 2017), fishes (Jafar et al., 2008; Liu et al., 2014; Zamri-Saad, 2018), and cattle (Zadoks et al., 2011; Lyhs et al., 2016; Sørensen et al., 2019). In humans, GBS is a leading cause of neonatal invasive disease, and a pathogen of immunocompromised adults and elderly people (Farley and Strasbaugh, 2001; High et al., 2005; Skoff et al., 2009). The epidemiology and clinical manifestations of GBS disease in humans continue to evolve, as exemplified by the recent emergence of hypervirulent GBS in adults without underlying comorbidities (Barkham et al., 2019). Phages and other mobile genetic elements (MGE) play an important role in the evolution of GBS, its adaptation to different hosts and niches and its virulence profile (Richards et al., 2019). In human isolates, a high prevalence of prophages has been associated with greater pathogenicity, particularly the ability to cause invasive infections (van der Mee-Marquet et al., 2006; Domelier et al., 2009; Salloum et al., 2010, 2011). A number of these phages carry genes associated with virulence and host adaptation, suggesting that lysogeny (the process of integration of temperate bacteriophages into the bacterial genome as lysogenic prophages), may play an important role in the biological success of the strains (van der Mee-Marquet et al., 2018). Likewise, host adaptation of a cattle-associated lineage is thought to have been driven by the acquisition of mobile genetic content, including prophages (Richards et al., 2011). This may include transfer of prophages between streptococcal species, as demonstrated between *Streptococcus pyogenes* and *Streptococcus equi* subsp. *equi* (Holden et al., 2009), *Streptococcus dysgalactiae* subsp. *equisimilis* (Davies et al., 2005), or *S. dysgalactiae* subsp. *dysgalactiae* (Suzuki et al., 2011), and suspected between *S. pyogenes* and GBS (Bai et al., 2013).

Considering the association of prophage carriage with virulence and host adaptation, there is a need for a method to screen isolates for the presence of prophages, and to classify these phages based on a reproducible typing scheme. Several bioinformatic tools for the identification of prophages in bacterial genomes are available on-line (Bose and Barber, 2006; Lima-Mendez et al., 2008; Arndt et al., 2016). Most of these tools, however, are based on databases of known prophage sequences, the composition of which can influence their performance and ability to detect prophages (Javan et al., 2019). If the database does not include phages that are specific to the bacterial species being examined, the program may only identify parts of the prophage structure and under-report the total number of prophages per genome (Javan et al., 2019). Prophage detection can also be hampered by assembly of the prophage sequence across multiple contigs (Jamrozy et al., 2017), which can happen in the case of short-read sequencing technologies such as Illumina (Bennett, 2004), particularly when draft assemblies are not closed. Short-read sequencing is currently the most widely used method of sequencing because of its accuracy of basecalling and low cost compared to most long-read sequencing techniques. Lastly, most prophage identification programs require at least some manual investigation of the output; this is impractical for large-scale genome studies, which are increasingly common.

A possible way to overcome these issues would be the adoption of a classification scheme based on host-specific prophage integrase types. This approach is already in use for other bacterial species, including *Staphylococcus aureus* (Goerke et al., 2009). These typing schemes are based on the concept that prophage integrases are site-specific (i.e., one type of integrase is usually found at only one chromosomal insertion site) through the recognition of attachment sites in the bacterial chromosome (*att*B) (Campbell, 1992), short nucleotide segments that are identical to attachment sites on the phage (*att*P). The *att*B corresponds to the insertion site where the phage recombines to become an integrated lysogenic prophage. Once the prophage is integrated, the *att* site is usually found at both ends of the prophage. Integrase-based typing schemes also exist for phage-inducible chromosomal islands (PICI), small molecular parasites that hijack phage packaging systems to be transferred to a new bacterial cell (Penadés and Christie, 2015; Mart́ınez-Rubio et al., 2017; Fillol-Salom et al., 2018). No bioinformatic programs specifically designed for the detection of these mobile genetic elements are available to date and manual inspection of whole genome sequence data is currently the only strategy for *in silico* identification of PICI.

A typing scheme for prophages based on full-prophage sequence diversity and insertion sites has been proposed for GBS (van der Mee-Marquet et al., 2018), however, a classification scheme based on site-specific integrases had yet to be developed for this species. Such a scheme would enable the rapid screening of large batches of full and draft genome sequences for the presence of prophages (based on integrase amino-acid sequences) and would be less affected by genome fragmentation. Additionally, as prophage integrases are thought to be site-specific, this typing method would allow for the unambiguous typing of the identified phages. To this end, we developed a GBS-specific prophage integrase typing scheme based on a large genomic dataset representing five continents and all major host species and clonal complexes of GBS.

## 2. Materials and Methods

### 2.1. Datasets Included in This Study

Screening for prophages and integrase genes, as detailed in the subsequent sections, was initially carried out using a publicly available dataset consisting of closed genome sequences obtained from NCBI (dataset 1, Table S1). The use of closed genomes ensured that prophage detection would not be affected by genome fragmentation and that complete prophage sequences could be identified for subsequent use. Dataset 1 comprised 69 closed genome sequences representing major and minor host species (human, *n* = 15; fish, *n* = 49; cattle, *n* = 2; camel, *n* = 1; frog, *n* = 1; unknown, *n* = 1) and five continents (Africa, *n* = 1; South America, *n* = 40; North America, *n* = 4; Asia, *n* = 22; Europe, *n* = 1; unknown, *n* = 1). Automated and manual screening of these isolates resulted in an initial database of prophages and integrase genes, similar to the existing *S. aureus* integrase typing scheme (Goerke et al., 2009).

To make the prophage and integrase database more comprehensive, a second dataset (dataset 2, Table S2) was subsequently screened, consisting of genomes that were of high quality, albeit not necessarily closed, and providing more in-depth coverage of major and minor GBS host species, geographic diversity, and GBS clades. Dataset 2 is a subset of 901 publicly available sequences included in the study by Richards et al. (2019) and includes all sequences with a maximum of 50 contigs (*n* = 503). Isolates with more than 50 contigs were excluded from analysis because high genome fragmentation can lead to sub-optimal performance of bioinformatic programs. As for dataset 1, isolates in dataset 2 originated from major host species (humans, *n* = 486; fishes, *n* = 8; and cattle *n* = 5) and minor host species (camel, dog, dolphin, and seal, *n* = 1 per species). Geographical origins were diverse (Africa, *n* = 1; the Americas, *n* = 353; Asia, *n* = 10; Europe, *n* = 117; Oceania *n* = 18; unknown, *n* = 4). Fourteen clonal complexes (CC) and 53 sequence types (ST) were represented in dataset 2 (Table S3), with the most well-represented being common GBS clades from humans (CC1, *n* = 260; CC17, *n* = 90; CC23, *n* = 56; CC12, *n* = 38; CC19, *n* = 29) or fishes (CC7, *n* = 6).

### 2.2. Screening of Genomes and Development of a Phage Integrase Typing Scheme

#### 2.2.1. Detection of Prophages and Integrase Genes in Dataset 1

**Figure 1:**
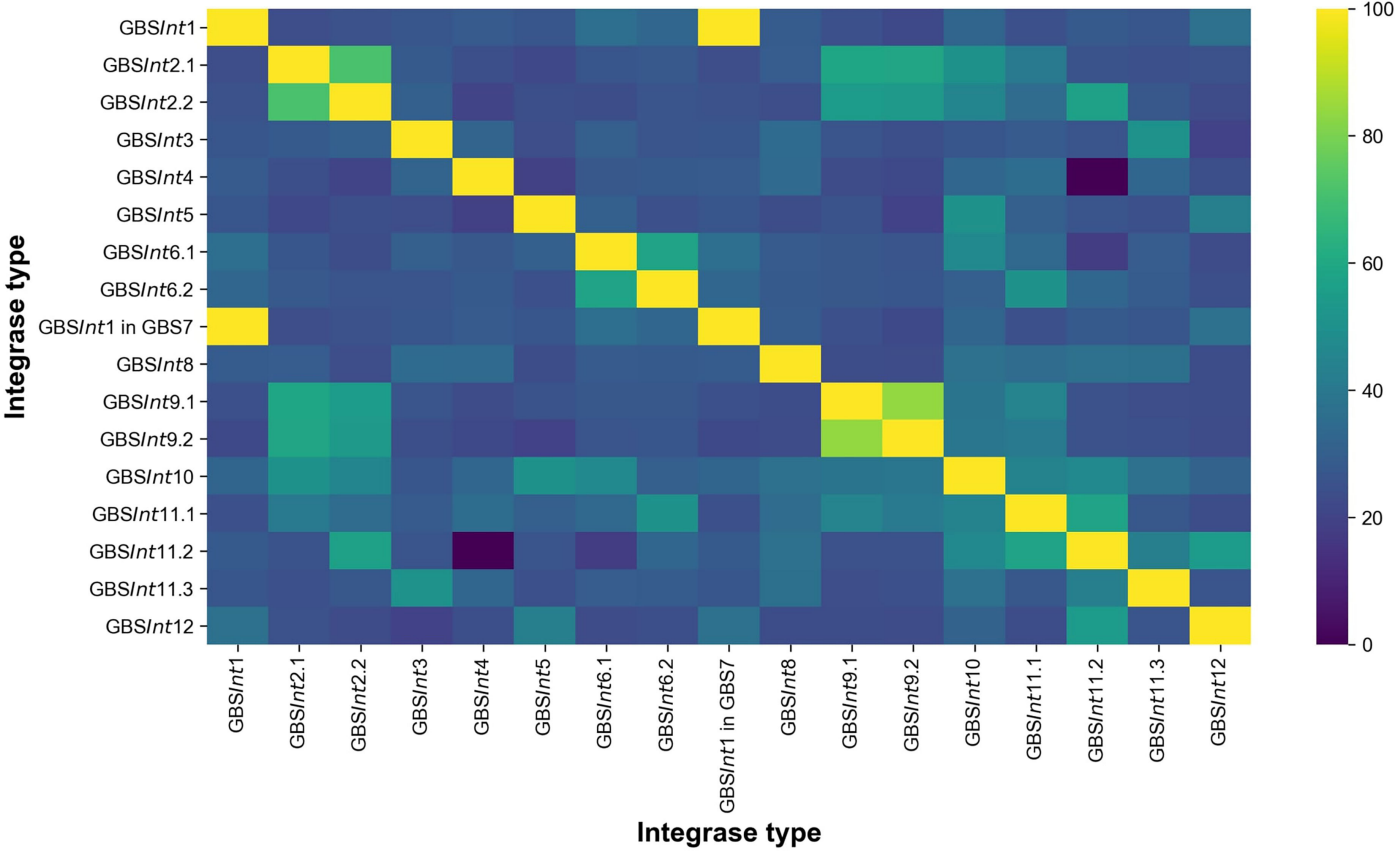
Heat-map showing the pairwise percentage of identities (% ID) at the amino acid sequence level between 16 integrase types identified in this work in Group B *Streptococcus* (GBS). GBS*Int*1 was found in two different insertion sites: GBS1 and GBS7.

To obtain the most complete database possible, and to assess agreement between methods, closed genomes from dataset 1 were analyzed with three methods, i.e manual screening of GenBank files, PHASTER, and PhageMiner. GenBank files were used for manual screening of phage sequences starting from genes annotated as “site-specific integrase,” “integrase,” or “recombinase.” Manual inspection was also used to identify putative PICI, as there are no specific bioinformatic programs available for the detection of these MGE. Details on how PICI manual inspection was carried out can be found in Supplementary Material, section 1.3. PHASTER (PHAge Search Tool Enhanced Release) (Zhou et al., 2011; Arndt et al., 2016) is a widely used web-based integrated search and annotation tool for phage display. PhageMiner (Javan et al., 2019) is a user-supervised semi-automated computational tool that enables the identification of prophage sequences within complete or draft bacterial genomes. It allows for rapid identification, user inspection and curation of phage sequences from large numbers of genomes and has been validated on streptococci (Javan et al., 2019). For our study, PhageMiner was run locally on GenBank files annotated with one of the recommended annotation tools for this program, RAST v2.0 (Aziz et al., 2008) or Prokka v1.11 (Seemann, 2014). Using complete prophages identified with the three approaches, a database of phage integrase types was built. Incomplete prophages, whether due to genome fragmentation or lack of essential genes such as the integrase, were not included in the analysis. Integrases were classified based on insertion site and percentage identity (% ID), using translated amino acid sequences, and numbered in order of detection. If a blastp (Camacho et al., 2009) comparison resulted in ≥90% ID (Figure 1, Figure S1) and ≥95% query cover (QC), integrases were considered to belong to the same type. When an integrase did not meet these thresholds but occupied the same integration site as an integrase that had already been classified, a subtype number was added (e.g., GBS*Int*2.1 and GBS*Int*2.2 represent integrases that both occupied integration site GBS2 but with <90% sequence similarity). The same set of prophages was identified with all three detection methods. Putative attachment sites were identified bioinformatically using blastn, through comparison of the site of integration in an empty genome (i.e., not harboring the prophage, chosen among closed genomes of ideally the same ST and host species) and the regions at both ends of the integrated prophage in a genome harboring the prophage.

#### 2.2.2. Detection of Prophages and Integrase Genes in Dataset 2

Because all methods identified the same prophages in dataset 1, only PhageMiner was used for dataset 2. It can be run locally, eliminating waiting time for server queues that may affect analysis speed for server-based programs like PHASTER, which is particularly relevant for large batches such as dataset 2. In addition, PhageMiner can generate annotated maps of putative prophage sequences, allowing for almost instantaneous inspection, and it can automatically store the extracted prophage sequences. For complete prophages identified in dataset 2, the integrase amino acid sequence was compared against the phage integrase database derived from dataset 1 using blastp to classify the phage integrase type, as detailed for dataset 1. PhageMiner searches often recognized phages as partial rather complete, even for full prophages, e.g., due to annotation of integrase genes and other prophage-related genes as hypothetical proteins. To allow for visual differentiation between partial and full prophages, the inspection window was widened, generally by 15 genes on either side. Closed genomes in dataset 2 (*n* = 25) were scanned manually for PICI identification, whereas draft genomes were screened for PICI presence with blast, searching for the integrase amino acid sequences of identified PICI.

### 2.3. Whole-Prophage and Integrase Gene Phylogenies

Two hundred eighty-two complete lysogenic prophages were detected in dataset 2. Using PhageMiner, all complete prophage sequences were extracted from the genomes (one complete prophage from 38% of genomes and two complete prophages from 9% of genomes) in dataset 2 and stored as GenBank files (*n* = 266). Prophages that straddled two contigs were excluded from the phylogeny (*n* = 16). Extracted prophages and 22 prophages identified by van der Mee-Marquet et al. (2018) were manually inspected and curated with Geneious v2020.1.2 (Biomatters Ltd, https://www.geneious.com). Sequences were reverse-complemented as needed to start with the integrase gene, and all integrase protein sequences were also stored separately. Multiple sequence alignments were performed for whole-prophage sequences and for integrase genes using ClustalW v2.1 (Thompson et al., 1994) with default settings (Gap opening penalty = 10, Gap extension penalty = 0.20). Approximately-maximum-likelihood phylogenetic trees were constructed from the sequence alignments using FastTree v2.1.11 (Price et al., 2010) using the Jukes–Cantor model with default parameters. Figures were edited using Inkscape (www.inkscape.org).

## 3. Results

### 3.1. Detection of Insertion Sites and Integrases Across the Prophage Phylogeny

**Figure 2:**
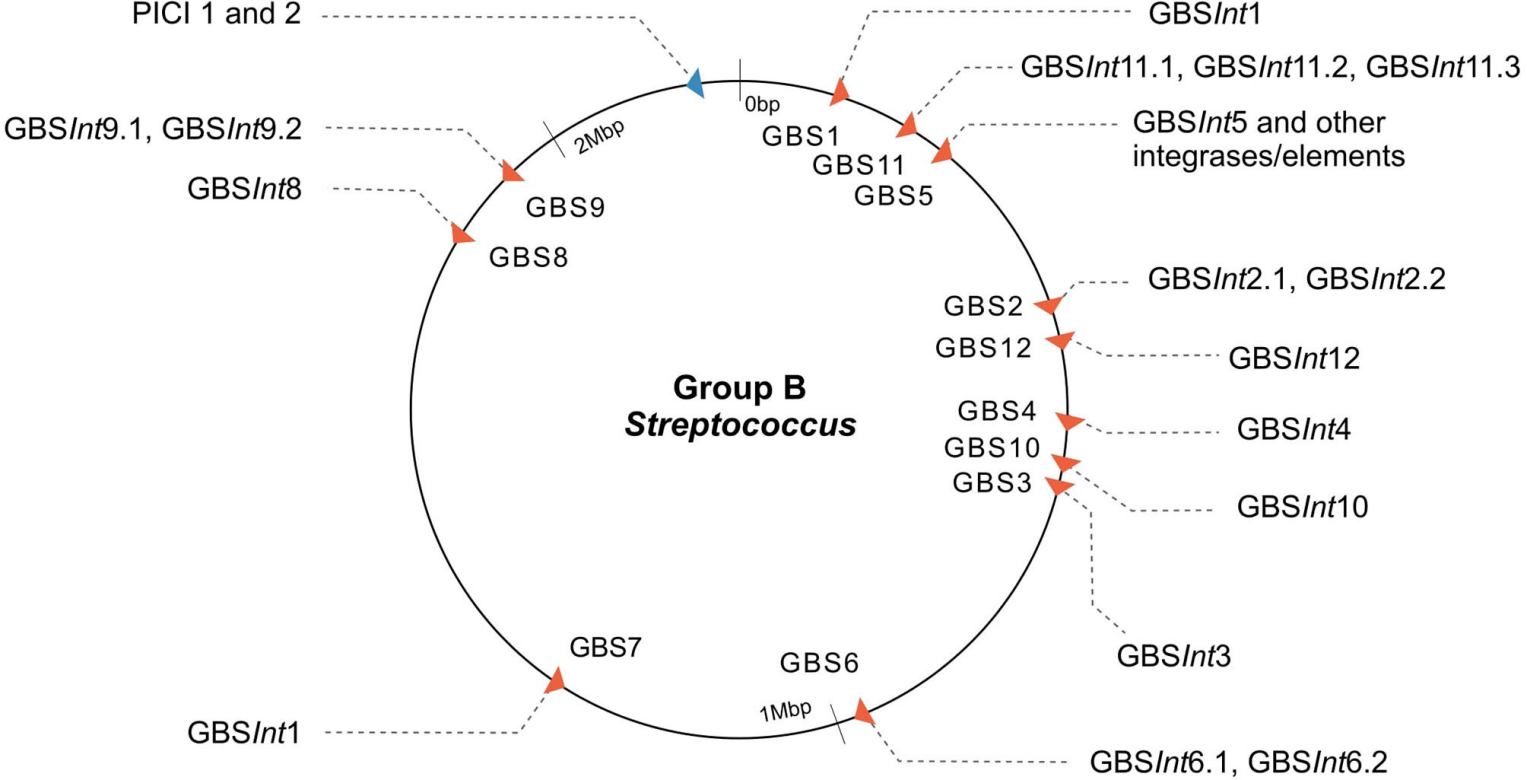
Map of the insertion sites of prophages and putative phage-inducible chromosomal islands (PICI) in Group B *Streptococcus* (GBS). Twelve phage insertion sites (red arrows) and sixteen integrase types were identified; phage insertion sites indicated with “GBS” and a progressive number based on order of detection are shown (integrase type sub-number indicates the different subtypes of integrases found at the same insertion site based on <90% similarity in the amino acid sequence). GBS5 integration site corresponds to the *rpsI* gene, a site of integration in common with ICE (Brochet et al., 2008; Ambroset et al., 2016) and PICI-like elements (this work). The putative PICI insertion site is displayed in blue and is the same for both PICI integrases (1 and 2). Arrows show the direction of packaging.

**Figure 3:**
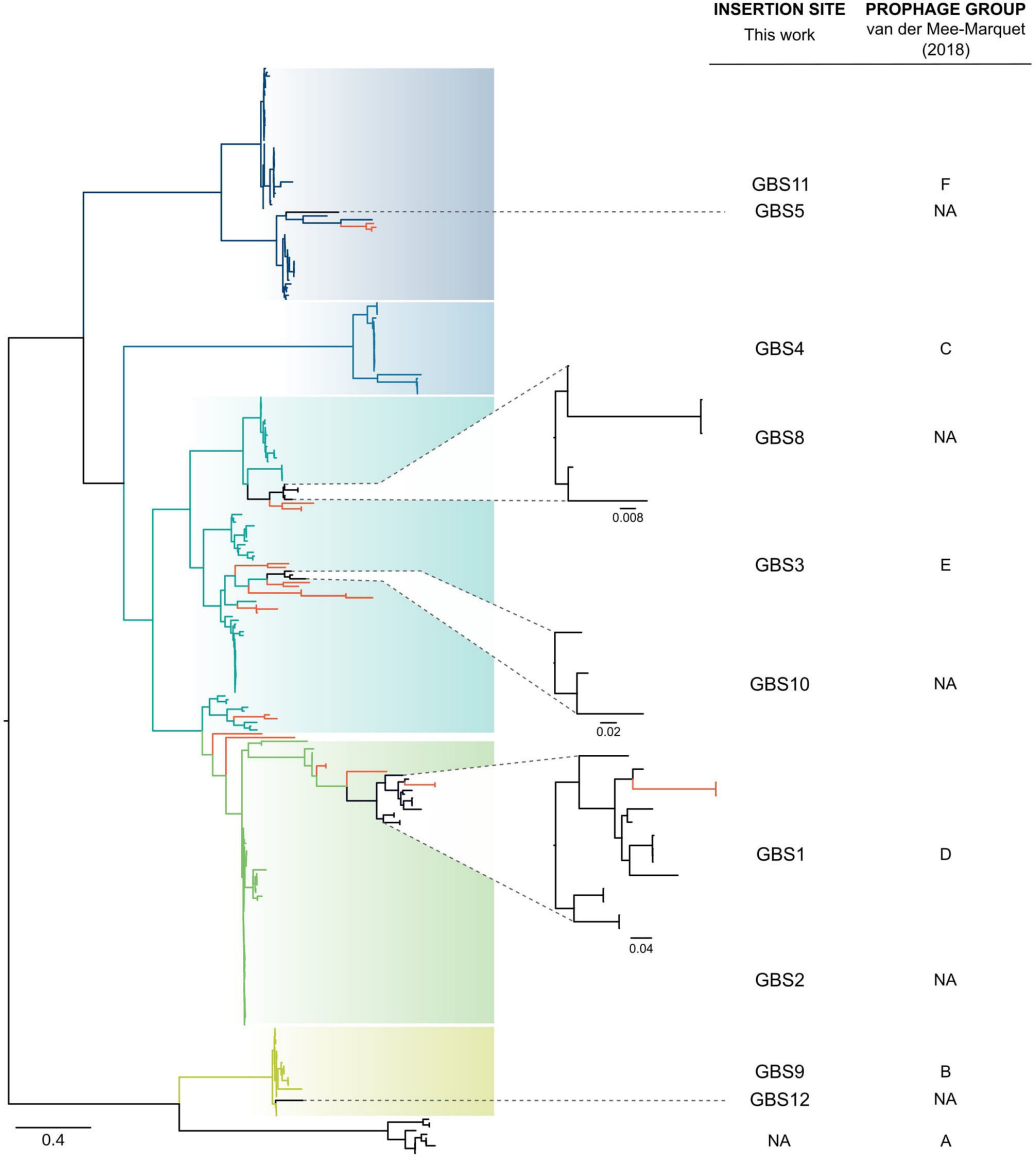
Approximately-maximum-likelihood phylogenetic tree of 266 complete prophages identified in group B *Streptococcus* (GBS) in this study and 22 prophages identified by van der Mee-Marquet et al. (2018). In most cases, full-prophage phylogenetic clusters are concordant with insertion sites and their corresponding integrase types or subtypes (GBS2, GBS3, GBS4, GBS9, GBS11, blue to green branches), with smaller clusters (GBS1, GBS5, GBS8, GBS10, GBS12, black branches) embedded in the larger ones. Some mismatches between prophages and their integrase type or insertion site and major clusters were identified (red branches), which is suggestive of integrase switching events. Tree was rooted at midpoint. NA, not applicable.

Twelve integration sites were identified and progressively numbered as GBS1 to GBS12. The 12 integration sites were occupied by 16 integrase types, implying that there were subtypes for some integration sites (Figure 2, Table 1). Ten integrase types were identified in dataset 1, with two additional types and four subtypes identified in dataset 2. The complete database of integrase types can be found in Supplementary Material, section 1.1 and at the GitHub online repository: (https://github.com/chcrestani/GBS_prophage_integrase_typing). Putative attachment sites (Table 1) were identified bioinformatically for twelve integrase (sub)types, but the search was inconclusive for five (sub)types. Mean prophage integrase length was 387 ± 48 AA (Table 1). Blastp comparisons of integrase type % ID and QC can be found in Table S4. Integrase types and subtypes predominantly clustered with their respective prophages in the whole-prophage phylogeny (Figure 3). Major prophage groups were located at insertion sites GBS2, GBS3, GBS4, GBS9, and GBS11. Phages with GBS*Int*2.2 (*n* = 60) were more common than those with GBS*Int*2.1 (*n* = 2). For completeness, one representative sequence of prophage type GBS*Int*11.3, which had been identified in other analyses, was added to the phylogeny of phages, and its integrase was added to the integrase phylogeny. Minor prophage groups (GBS1, GBS5, GBS8, GBS10, GBS12) branched out from within major clusters. For some prophages, a mismatch between their integrase type and the integrase type of the surrounding prophage cluster was observed (red branches in Figure 3, Figure S2). This included all GBS6 prophages (GBS*Int*6.1 and GBS*Int*6.2), which were distributed across multiple branches of the prophage clades associated with GBS2 and GBS3 (Figure S2). GBS*Int*4 was associated with its own monophyletic phage clade and integration site, GBS4, but was also found on branches of the prophage clades associated with GBS2, GBS3 and GBS11. Likewise, GBS*Int*2.1 and GBS*Int*2.2, GBS*Int*3 and GBS*Int*11.1 were associated with their own clade and integration sites (GBS2, GBS3, and GBS11, respectively), as well as being detected in other prophage clades, i.e., GBS2 and/or GBS3. The integrase phylogeny (Figure S3) showed defined clusters, with varying levels of diversity within clusters. Integrases located at the same insertion site generally formed monophyletic clades, with the exception of GBS*Int*11.3, which was more closely related to GBS*Int*3 than to GBS*Int*11.1 or GBS*Int*11.2.

**Table 1:** Insertion sites of 16 integrase types for phages identified in Group B *Streptococcus* isolated from across multiple host species and countries.

**Insertion site**	**Phage Integrase type**	**Phage integrase length (AA)**	**Gene**	**Putative attachment site**
GBS1	GBS*Int*1	369	*comX* (sigma-70 familyRNA polymerase sigma factor)- 3′ end	*att*L TTTTTTGTTATAATATAA**GA** *att*R TTTTTTGTTATAATATAA**TA**
GBS2	GBS*Int*2.1	360	tRNA methyltransferase—3′ end	ATCCCCTCCTCTCCTTTAAT
	GBS*Int*2.2	363		
GBS3	GBS*Int*3	368	*rplS*—3′ end	*att*L GATTCC**G**GCAGGGGACAT *att*R GATTCC**A**GCAGGGGACAT
GBS4	GBS*Int*4	389	HU, histone-like DNA-binding protein	CTCTTAAAGACGCTGTTAAATA ATTCGTCTAGAAAAACCTTGTC ATATCAATGTTTATTGATAGCGAC AAGGTTC
GBS5	GBS*Int*5	331	*rpsI*—3′ end (within ICE)	–
GBS6	GBS*Int*6.1	366	CatB-related O-acetyltransferase—5' end	TGGAGCCGGTGGGAGT
	GBS*Int*6.2	366		
GBS7	GBS*Int*1	369	*hylB*—5′ end	*att*L TTTTTTGTTATAATAT**AAGA** *att*R TTTTTTGTTATAATAT**GAGA**
GBS8	GBS*Int*8	382	YbaB/EbfC family nucleoid-associated protein	TTTTGCATATTCATCATA
GBS9	GBS*Int*9.1	360	*nhaK* (sodium/proton antiporter)—3′ end	AAGGCGGTAGACGGATTTGAA
	GBS*Int*9.2	359		
GBS10	GBS*Int*10	476	DNA-binding protein WhiA—3′ end	-
GBS11	GBS*Int*11.1 GBS*Int*11.2 GBS*Int*11.3	489 486 367	*gspF* or *gspF*+competence proteins/type II secretion system proteins—3′ end	CTTTTAGAATGTTTGGTA–
GBS12	GBS*Int*12	386	5-formyltetrahydrofolate cyclo-ligase—5′ end	-

*With the exception of GBSInt1, integrases are site specific. Putative attachment sites (att) are shown when known. For att sites that differed slightly at the two ends of the lysogenic prophages, left and right (attL and attR) sequences are specified and differences are highlighted in bold*.

### 3.2. Insertion Site Peculiarities and PICI Identification

GBS*Int*5 was identified in GBS5 (*rpsI* gene) in genome QMA0323, where the full prophage is present, preceded and followed by other genes with signatures of an ICE (integrative conjugative element) (Figure S4). By contrast, in genome FSL_S3-026, integrase GBS*Int*5 is present as a singleton, i.e., not followed by a full prophage. Rather, it was found inside what was classified as a putative ICE (~67,000 bp) by ICEFinder (Liu et al., 2018). This larger ICE showed partial similarity with a region of ~9,000 bp found after the prophage in QMA0323.

The label GBS*Int*7 is not used because the site-specific integrase at insertion site GBS7 was identical to GBS*Int*1 at insertion site GBS1 (Figure 1, Table 1). GBS*Int*1 at site GBS7 was only observed in this location when the GBS1 site was occupied by a prophage and it was uniquely observed in ST283, the only known hypervirulent GBS in human adults (Barkham et al., 2019).

At insertion site GBS11 (Table 1), the full prophage immediately followed *gspF* (*n* = 18 genomes), or it was separated from *gspF* by a few genes encoding small proteins (*n* = 17 genomes). The latter included competence proteins, type II secretion system proteins, and hypothetical proteins (Figure S5). There was no clear correlation between the integrase subtype (GBS*Int*11.1, GBS*Int*11.2) and any of these GBS11 site variants, but there was correlation between prophage subcluster and integrase type (Figure S6). For 26 prophages with either GBS*Int*11.1 or GBS*Int*11.2, it was not possible to assess the integration site because the prophage was found at the end of a contig.

**Figure 4:**
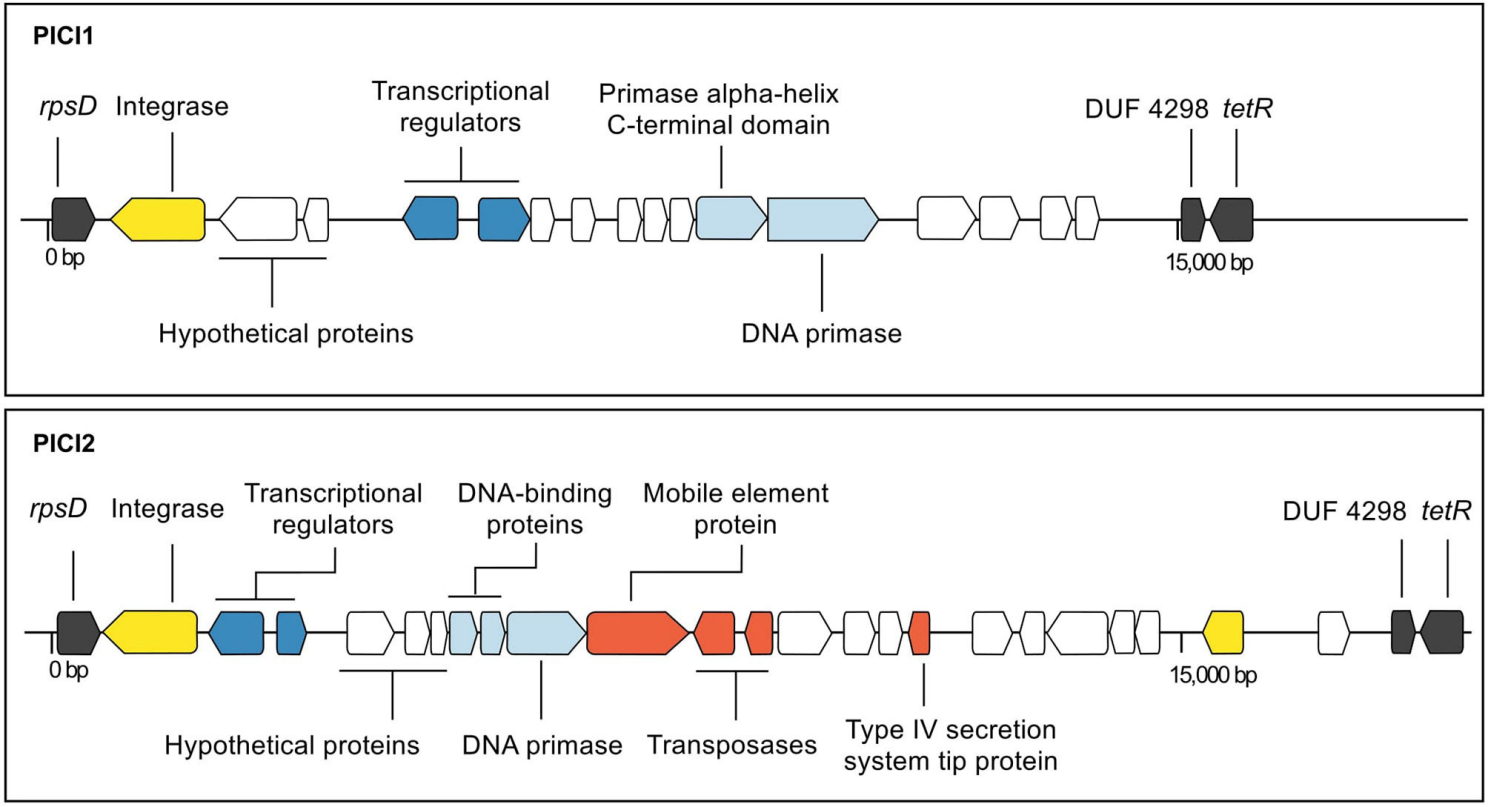
Annotated maps of genes in putative phage-inducible chromosomal island (PICI) 1 (strain 09mas018883) and PICI2 (strain ILRI005) of group B *Streptococcus*. The integration site is the same for both integrases (*rpsD*). Genes are color-coded based on function (black: chromosomal genes; yellow: site-specific integrase; dark blue: genes involved in lysogeny; light blue: replication genes; white: hypothetical; red: other genes).

In addition to prophages, two putative PICI sequences (PICI1 and PICI2) were identified using manual screening (Figures 2, 4). Both integrases were 398 AA long and shared the same integration site (*rpsD* gene). Amino acid sequences for PICI1*Int* and PICI2*Int* can be found in Supplementary Material, section 1.2. PICI2 was uniquely identified in dataset 2. PICI-like MGE were also detected in the integration site corresponding to the *rpsI* gene, i.e., in the same location as GBS*Int*5 (Figure S7). However, it was not possible to classify these PICI-like elements with certainty, as they could have been fragments of other elements such as prophages or ICE (see section 4).

### 3.3. Detection of Prophages Across Host Species, Countries, and GBS Clades in Dataset 2

To create as complete an integrase database as possible, GBS genomes representing a wide variety of host species, countries, and GBS clades were included in the analysis. The study was not designed to be an epidemiologically representative survey of prophage or integrase distributions, so calculation of prophage prevalences is not meaningful, but some qualitative observations about the association with genome origin can be made.

Complete prophages were detected across isolates from most host species, including 47% of human GBS genomes (*n* = 230 out of 486 isolates, with a total of 274 complete prophages) and three fish GBS genomes (Figure S8) but with the exception of bovine and canine GBS genomes (*n* = 5 and 1, respectively). PICI were also detected across GBS from most host species, with PICI1 found in a total of 328 GBS genomes from humans, fish, cattle, a dog and a dolphin, and PICI2 found in a camel GBS genome from Kenya.

**Figure 5:**
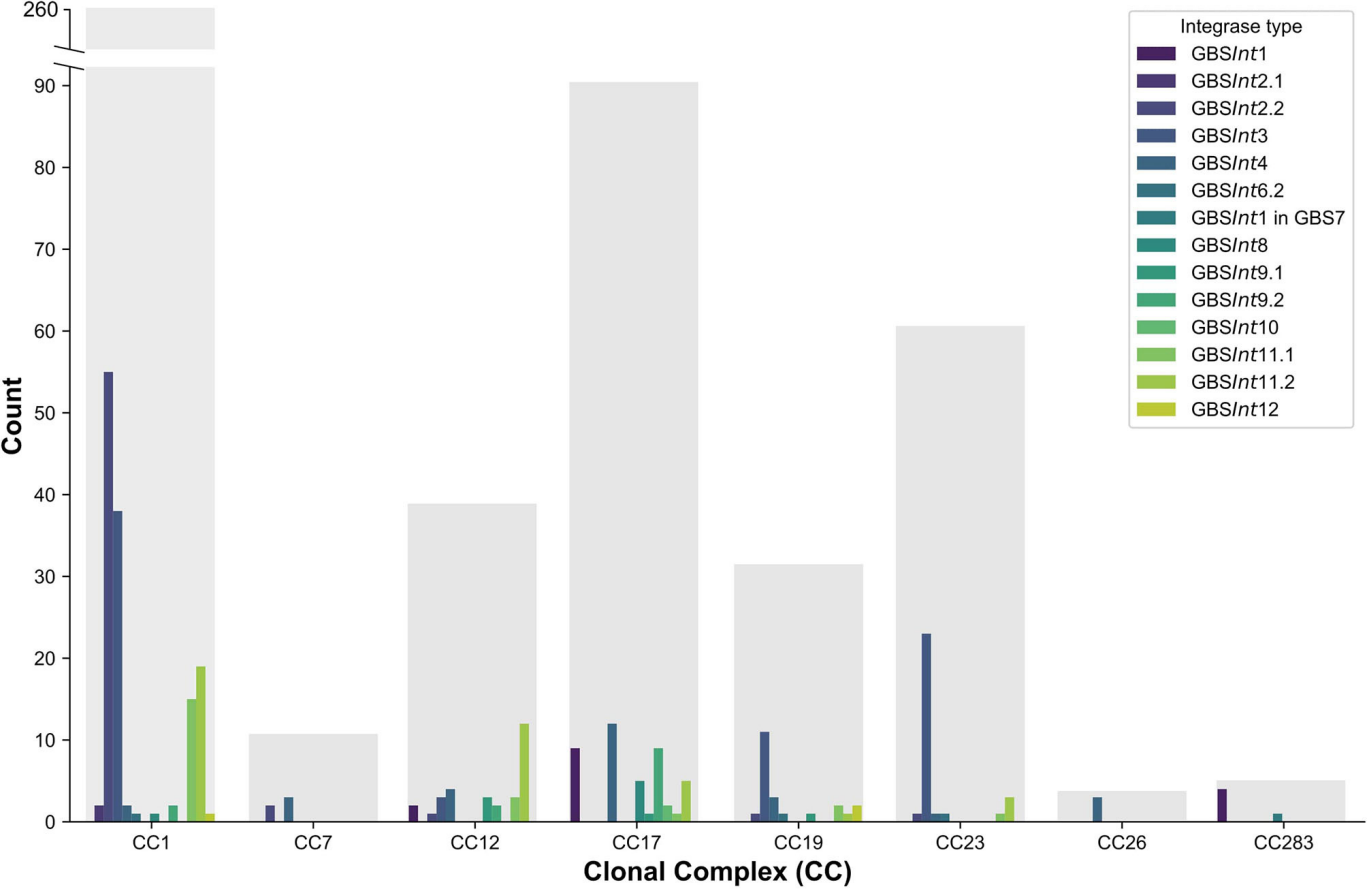
Distribution of complete prophages classified based on their integrase types (GBS*Int*1 to GBS*Int*12) in a publicly available dataset of 503 Group B *Streptococcus* (GBS) genome sequences (dataset 2) comprising a global collection of isolates from seven hosts species. Results for major clonal complexes (CC) are shown. Gray bars show the total number of genomes per CC.

Prophages and integrases were detected in most GBS clades, with the exception of certain clades represented by 3 or fewer isolates (CC22, CC67, and CC130, Table S5), and the majority of integrases were detected in multiple clades (Figure 5). The number of integrase types per CC ranged from 1 to 10 (Figure 5). All major ST in dataset 2 (ST1, ST17, ST19, ST23, ST459) harbored at least four prophage types (Table S6).

Complete prophages were identified in GBS isolates from all continents except for South America. The number of discovered prophages tended to reflect the total number of genomes per continent, whereby more prophages were detected in continents with more genome sequences (Table S7).

## 4. Discussion and Conclusions

We describe the development of a typing scheme for GBS prophages based on site-specific integrase genes and insertion sites, similar to the scheme used for prophage typing of *S. aureus* (Goerke et al., 2009; Valentin-Domelier et al., 2011; Jamrozy et al., 2017). The scheme is intended for detection of putative prophages through BLAST searches (tblastn) using one or several genome assemblies (nucleotide sequences) as subject sequences, and the database of integrase protein types presented here as query sequence as detailed in the section 2, Supplementary Material, and online (https://github.com/chcrestani/GBS_prophage_integrase_typing). This approach enables the rapid screening of large datasets of complete and draft genomes for the presence of GBS prophages, overcoming some of the limitations associated with existing phage detection programs, and enabling detection of phage content in fragmented genome assemblies. Additionally, BLAST-based searches of integrases can be performed by those with little computational experience, as BLAST is available as an online platform.

Phage integrase typing agreed with full-length prophage genome-based phylogenetic clusters, with a few exceptions. This is reminiscent of the relationship between the GBS whole-genome phylogeny and capsular serotypes, where serotypes tend to match phylogenetic clusters but capsular switching may occur (Martins et al., 2010; Bellais et al., 2012; Neemuchwala et al., 2016). We propose that integrase switching may also occur, leading to “mismatches” between prophage genome phylogeny and integrase phylogeny, and conferring to the prophage the ability to integrate in a different location in the GBS genome. This genome plasticity may impact on the function of the prophage, and on packaging of GBS genome content. There is growing evidence that prophages contribute to emergence, niche adaptation and spread of virulent GBS, especially in CC1 and CC17 (van der Mee-Marquet et al., 2018; Renard et al., 2019; Jamrozy et al., 2020). This may include transfer of prophage content between GBS from different host species, in agreement with the detection of prophage types and integrase types across GBS from different host species in our dataset. We discovered a potential contribution of prophages to the emergence of hypervirulent ST283, which has recently been recognized as a major cause of adult invasive disease in Southeast Asia (Rajendram et al., 2016; Kalimuddin et al., 2017; Barkham et al., 2019). Contradicting the dogma that phage integrase genes are site-specific (Frost et al., 2005), the integrase at insertion site GBS7 (5' end of *hylB*), was identical to the integrase at GBS1. Prophages in GBS7-*hylB* were only present when GBS1 was also occupied by a prophage, and they were unique to ST283. The virulence gene *hylB* codes for hyaluronate lyase, an enzyme that degrades extracellular matrix components and is believed to contribute significantly to invasion (Herbert et al., 2004; Maisey et al., 2008). We hypothesize that this prophage plays a role in regulation of the transcription and expression of *hylB* and contributes to the hypervirulence of ST283.

Our analysis of 572 GBS genomes extends previous work by van der Mee-Marquet and colleagues—who identified prophages in 14 GBS genome sequences to subsequently screen by PCR a larger collection of isolates (*n* = 275)—by increasing the number of known prophages, insertion sites and integrase types. Our major full prophage clades matched previously defined prophage groups (Prophage group B = GBS9, C = GBS4, D = GBS1, E = GBS3, F = GBS11) (van der Mee-Marquet et al., 2018), whilst other clusters, including those located at GBS2, GBS5, GBS8, GBS10, and GBS12, are described here for the first time. As the GBS genome database expands, the typing scheme will need to be updated with emerging integrase types and subtypes. This is also illustrated by results for insertion site GBS11 (3' end of *gspF*), which is located within an operon involved with host competence (*com* operon). GBS11 had previously been classified as two separate insertion sites, F1 and F2, based on variations observed between three prophage genomes at this site (van der Mee-Marquet et al., 2018). Based on our analysis, the bifurcation of this group of prophages, which was also observed in our whole-prophage phylogeny, correlated with different integrase types (GBS*Int*11.1 and GBS*Int*11.2), rather than with different insertion sites. In many cases the insertion site for those integrases could not be confirmed because they were located at the edge of a contig. This suggests that sequence assembly tools struggle to assemble this region of the GBS genome, an issue that could be overcome by long read sequencing.

Our typing scheme does not include type A prophages (van der Mee-Marquet et al., 2018) because they are defective rather than whole prophages, lacking the integrase gene. Although this could be considered a false negative result in our typing scheme, lack of an integrase gene renders type A prophages incapable of horizontal gene transfer so that they can only be spread through vertical transmission, limiting their contribution to the evolution of virulence or niche adaptation. Based on integrase typing, false positive results may also occur, as demonstrated for GBS*Int*5, which was found once as part of a full prophage (isolate QMA0323, piscine ST261; Kawasaki et al., 2018) and once as a singleton within a larger ICE (isolate FSL S3-026, bovine ST67; Richards et al., 2011). A BLAST search of genomes with more than 50 contigs showed the presence of GBS*Int*5 as a singleton within an ICE rather than as part of a full prophage in nine bovine GBS genomes from bovine-associated lineage CC67 (Richards et al., 2019). This phenomenon was only observed for GBS*Int*5, possibly because its insertion site, *rpsI* (30S ribosomal protein S9), is a hotspot for recombination of ICE in streptococcal species (Brochet et al., 2008; Ambroset et al., 2016). When this integrase is identified within a dataset, further analyses need to be performed to determine whether a full prophage is present. The GBS5 insertion site also contained PICI-like elements. Because of these multiple integration events, PICI-like elements in this position could not be classified with certainty, as they could have been fragments of prophages or ICE.

PICI1 and PICI2, which are reported here for the first time, were integrated into *rpsD*, which encodes 30S ribosomal protein S4. This gene had previously been described as the site of integration of an *S. agalactiae* chromosomal island (SagCI) in 3 of 9 complete GBS genomes (Nguyen and McShan, 2014), but not as a PICI. GBS differs from other members of the pyogenes group, including *S. pyogenes, S. canis, S. dysgalactiae* subsp. *equisimilis*, and *S. parauberis*, in that their chromosomal islands are primarily integrated in *mutL* rather than *rpsD*, which may affect their functional impact. The structure of both PICI1 and PICI2 includes typical PICI features such as transcriptional divergence, a size of around 15,000 bp, and presence of a DNA primase (Mart́ınez-Rubio et al., 2017), whereas the variable portion of their organization and content resembles the structure of SpnCIST556 in *S. pneumoniae* and SpyCI6180 in *S. pyogenes*, respectively (Penadés and Christie, 2015). Streptococcal PICI may have roles in gene regulation (Nguyen and McShan, 2014) or gene transfer (Mart́ınez-Rubio et al., 2017). The high prevalence of PICI1 across GBS genomes from different host species, geographic origins and clades suggests that its function warrants further investigation. By contrast, PICI2 was exclusively found in one isolate from CC609, which is a camel-specific clade from East Africa (Fischer et al., 2013). Further research and experimentation would be needed to understand if and how PICI play a role in GBS evolution and virulence.

In summary, we propose a new typing scheme for rapid prophage identification in large datasets of GBS genomes based on site-specific integrase types and their insertion site. This method provides a practical way of identifying potential prophage presence with a BLAST-based approach in full and draft genomes, overcoming detection issues related to genome fragmentation and making it user-friendly for researchers with any level of computational experience. We show that multiple prophages and integrase types occur across GBS from a wide range of host species, geographic origins and clades, and that a secondary prophage was uniquely present in hypervirulent GBS ST283. In addition, we report the high prevalence of putative PICI in GBS, opening up a new area of GBS research.

## Data Availability Statement

All datasets presented in this study are included in the article/Supplementary Material.

## Author Contributions

RZ and CC conceived the study. CC conducted the data analysis with guidance from TF. CC drafted the manuscript. All authors edited the manuscript.

### Conflict of Interest

The authors declare that the research was conducted in the absence of any commercial or financial relationships that could be construed as a potential conflict of interest.
