# Supplementary material for: Development and Application of a Prophage Integrase Typing Scheme for Group B Streptococcus
Source: Front Microbiol. 2020 Aug 31;11:1993. doi: 10.3389/fmicb.2020.01993 (PMC7487436; doi:10.3389/fmicb.2020.01993)
Supplement: Supplementary file 1 [file Data_Sheet_1.pdf]

frontiers

Supplementary Material

1 SUPPLEMENTARY DATA

1.1 Database of amino acid sequences of prophage site-specific integrases identified in this study.

GBS*Int*1 (same as integrase at GBS7)
MIEKYTKKDGTTAYRLRAYLGVDPMTGKQVRTTRQGFKTEREAKRAEVKLIDDFQRQGAWKSNDKTTFDDVAKLW
FEQYRNTVKPSTFLVNQNYYKTILKPHLGQLQMTKITVMICQKFVNCLSRYSGYRLYLSLANRIFKFAVNLGIIDNNPM
SKTLRSKCTYKNMDTLTKKYYTKEELNAFLRIVEAEETLEMRLIYRLLSYGGFRIGELIALKDTDFDFRNNTISITKTIA
YTKEGWAVQSPKTKKSNRTISMDAETMTLAKLYIKQSIKPLHGSFKLFNFASDTVRKRLDRFILKHGLKRIPHGFRHT
HASLLFEAGIPAKIAQERLGHAKIAITMDLYTHLSKKSKDNVADKLAELVAI

GBS*Int*2.1
MASYRKLDSGWEYRIIYKDINGIRREKSKRGFSTKTLAKAAAVKAEREINSTDTELLDTTFYDYSIQWAEVYKRPHVT
AKTWQTYSKNFKHIKHYFGNMKVKDITHTFYQKVLNEFGEIVAQQTLDKFHYQVKGALKSAVRDGIIRYNVADGAI
VKSQVAKKSKEEKFLEESDYLNLIEVSKDKIKYASYFTVYLIAVTGLRFAEVQGLTWNDVDFDNGFLDINKSFDYSIS
QRFAPTKNEQSIRKVPIDLNTIDILKEYKDNYYQPNKLGRICYGASNNATNKAIKLTTGKPYPTNHTLRHTYASYLIMQ
GVDLISISQLLGHENLNITLKVYAHQLDKLKEKNDKVIKDIFYNL

GBS*Int*2.2
MAFYRKLGSGWEYRITYRDSQGKKREKSKRGFKTKTLAKVAAQQAEIDLNTMTADLLDITVLDYNRRWADIYKKPH
ITAKTWQTYTKNFKHIEHYFGTRKLKSITHTFYQQVLNDFGEKVAQQTLDKFHYQIKGACKMAIRDGIIRDNFADGAI
VRSQKPVKEESEKFMEESEYLTFIKVAKSKVKYPSYLTTYIIAVTGLRFAEVQGLTWKDIDFDNGYIDINKTFDYSISQN
FGPTKNEQSIRKVPIDKNSLELLRNFKSNYYQDNKLDRICFGASNNATNKVIKRVTGRNLTNHSLRHTYASYLIAQGV
DLISVSKLLGHENLNITLKVYAHQIESLKEKNDHQVKNIFQNLKFDG

GBS*Int*3
MRYKTMWIEELANGKFKYIERYTDPLTNKYKKVSVTLDKNSSQAQKKAGLILQEKIEDRLAIRNHSEMTYGELKKEY
LKQWIPTVKDSTKRGYLVSDSHIATVLPDDTIINKLTKRDIRLIIDKLLKHNSYHVTHKCRKRLHAIFSYAIQMDYMTS
NPTENVLVPKPKDDYKPEKVLYLTSNEVYDLCNRMIDNDEQTLADIVLFMFLTGVRYGELACLTYDKIDFENKEILIN
ATYDFNTREITTTKTKKSTRKISVSDNILDIVNKQKKTSSFVFPNSNGVPILNAYINKRLKIYGDYHTHLFRHSHISFLAE
KGIPLNAIMDRVGHSDPKTTLSIYSHTTVNMKEIINKQTAPFVPFLKPE

GBS*Int*4
MRQKSMWSEKHKSGKVNFVERYKDPYTNKWKRTSVLMEKDTPRIRKEAQRILEAKIADIVRKLQTSDMLFTNLIDEW
WIFYQQEIKRSSIVTLKGNIREIRAEFGINIPVVNIDPRYVQNYLDNLDCSRNKKERNKSMLNLIFDYAVSLDIIKDNPAR
RAKLPKIKKTLNDWKKIEEKYLEEEEIKRLLKELFRRPSTRRLGLLSEFMSLNGCRIGEAISIEPDNIDFKNKTLQLHGT
YDRTNGYINGEKTSPKTLASYRETIMTKREMEIIQELEFINELEKNTNPRYRDMGYIFTTRNGVPIQINSFNLALKKANE
RLEQPINKNITSHIFRHTLVSRLAENNVPLKAIMDRVGHADAKTTVQIYTHITKKMKSNIADIMENY

GBS*Int*5
MKDKIITQVVSIMAEQLTMEQLEQLERVLAANLANVVMTENVSKVDETSNPKLLHLFISAKRIEGCSEKSLKYYKMV
IEKMVAELDKPIRQISTSDLRTYLANYQKERQSSKVTIDNMRRIFSSFFSWLEDEDYILKSPVRRIHKIKTDKVIKETLSD
ESLELLRDTCDNIRDLAMIDLLASTGMRVGELVRLNREDINFHERECLVFGKGNSERIVYFDARTKIHLINYLDSRKDD
SSALFVSLAYPYDRLMIGGVETRLREIGKRANLQKVHPHKFRRTLATRAIDKGMPIEQVQHLLGHVKIDTTMHYAMV

NQANVKNSHRKYIG

GBS*Int*6.1
MRIESYKKKNGTTAYKFLLYAGYVDGKRKYIRRSGFSTRQSARAALINLQAELEKPKSSMTFGMLTKQWLKEYEKT
VQGSTYLKTERNINKHILPKLDKVTIGDINPLLVQNLTEEWCSQLKYGGKILGLVRNILNLAVRYGYISNNPALPITAPK
IKRERKTGNNFYTLNQLKQFLELVEKTDNIEKIALFRLLAFTGIRKGELLALTWDDLNRNTLSINKAVTRTQTGLEIDV
TKTKSSDRLISLDDETLEILQQLHETFPSSTFMFQSESGGIMTPSLPRKWLLQIIKGTDLPQITVHGFRHTHASLLFESGL
SLKQVQHRLGHGDLQTTMNVYTHITQSAIDDIGTKFNQFVTNKQLN

GBS*Int*6.2
MQIESYQKKNGTTAYRFRIYIGVIDGKKKYIKRSGFTSKKIAKQALMNLQQEIENPESKSTMLFHELTNLWLNNYEKT
VQSSTYLKTKRNIENHILPSLGNYPIKDLTPLIIQKYADEWAVKLKYSSKIVGTVRNILNYAVKFQYIPSNPSDPVTTPK
IKRTINKKKDYYNKDELKEFMQLVYDTDNIDIIATFRLLAFTGLRKGEMLALTWKDYRNGTIDVNKAIARDITGEYVG
PTKNKSSERLISLDPETINILDELHETYPKTKYILESTAGRWISPTQPRRWLLQILSNSKSRLEPIRIHGFRHTHASLLFESG
LTLKQVQYRLGHEDLKTTMNTYVHITESAKDDIGTKFSQYIDF

GBS*Int*8
MWHEEQANGNIKFIEYYKDPYTGKRQRAYVTLDRYTKQSETKARRLLNEIIECRIKSSGDQFVRFGQLVEEWKTSHS
KTVKARTMKVYRHPIEKIKDFIGDDVLVKNIDARLLQKFIDYLKDRYSDNTINLIKQPLNMMLNYAVRMEYIMSNPM
KNVVTPKRKKMSKKQFEDKYLETEQNQKIIEQLRDPIYGNHIANFSEIIFLTGMRPGELLALRWDHIDFEKLKIKIEYTL
DYTTNGHANAELGSVKNDGSYRTIDIPLRVKEMLVEELNYQNTNDLRSDFVFITNKGKHLSINTINRRIKKTSEKLYG
IVITSHSFRHAHITLLAELGIPLKSIMDRVGHTDVNTTIKVYTHATDKIGKQMMDKINKFVPIQSL

GBS*Int*9.1
MASYRKRENGLWEYRISYKTIDGKYKRKEKGGFKTKKLAQAAAIEIEKKLTQNILTNDEVTLYDFVKTWSEVYKRPY
VKDKTWETYSKNFKHIKNYFQELKVKDITPLYYQKKLNEFGEKYAQETLEKFHYQIKGAMKVAVREQVVTFNFAEG
AKVKSQVEPKNEEEDFLEEREYKALLALTRENIQYVSYFTLYLLAVTGLRFSEAMGLTWSDIDFKNGILDINKSFDYS
NTQDFADLKNESSKRKVPIDSNTIDILREYKKNHWQANIKNRVCFGVSNSACNKLIKKIVGRKVRNHSLRHTYASFLI
LNGVDIVTISKLLGHESPDITLKVYTHQMEALAERNFEKIKNIFLVA

GBS*Int*9.2
MAYYRKRDNGWEYRISYKDESGKFRQKSKSGFKTKKLAQAAARDIEKKLSQNILTDGEVTLYDFVKTWSEVYKRPY
VKDKTWETYTKNFRHIKTYFKDIKVKDITPLYYQKRLNEFGEKYAQETLEKFHYQIKGAMKVAVREQVIHFNFADDA
KVKSQIESRAEENDFLEESEYKALLSLTRENIQYVSYFTLYLLSVTGLRFSEVMGLTWNDVDFKNGILDINKAFDYSNT
QDFCDLKNNPSERKVPIDRKTIEILYVYRQNYWQANIKNRICFGVSNSACNKLIKKIIGRPVRNHTLRHTYASFLILNGV
DIVTISKLLGHESPDITLKVYSHQMEALAERNFEKIKNIFLAS

GBS*Int*10
MRKVAIYSRVSTINQAEEGYSITGQIDSLTKYCDAMGWVIYKNYSDAGYSGGKLERPAISELIEDGKNNKFDTVLVYK
LDRLSRNVKDTLYLIKDIFTKNNIHFVSIKENIDTSSAMGNLFLTLLSAIAEFEREQIKERMQFGVMNRAKSGKTTAWK
TPPYGYTYDKENKVLLLNEFEATNVKQIFNMIVAGHSIMSITNYAKEHFAGNTWTHVKIRRILENETYKGLVKYREQT
FAGNHDAIIDEELFTKAQLALDKRTNSQNNTRPFQGKYMLSHIAKCGYCGAPLKVCTGRPRVDGTRRQTYVCVNKT
ESGAKRGVNNYNNNKVCNSGRYEKSCVEKYVINELSKIQHDKEYLEKMKNNSKKVDVSSLKKEIKSIDKKINRLNDL
YVNDFISLSKLTEEIKKLNKLKEGYHKTIKLNYVENKNEDVISTLVNNIDISKSSYDVQSRIVKQLVDRVEVTTDNIDIIF
NF

GBS*Int*11.1

MNKVAIYVRVSTTMQAEEGYSIDEQIDKLKSYCKIKDWTVYDIYKDGGFSGGNIERPAMERLISDAKRKKFDTVLVY
KLDRLSRSQKDTLFLIEEVFDKNDISFLSLNESFDTSTAFGKAMIGILSVFAQLEREQIKERMLLGKIGRAKTGKSMMFS
KVSFGYTYDKLKDELVVNQAESIIVRKIFDAYLGGLSLNKLRDYLNNNGIYRGDKPWNYQGLRRILSNPVYIGMIRYR
EEIYPGNHKAIIDIDDYNKTQEEIKKRQIKALEFSNNPRPFRSKYMLSGIAKCGYCGTPLQIILGSKRKDGTRNMRYQCI
NRFPRNTKGVTIYNDGKKCESGFYEKADIEEFVINEIRSLQINYNKLDAMFDRHPTVNSDDIKKQIITLDNKLKRLNDL
YINNMIELDDLKKQTQSLRKQKTILEDELLNNPAITQEKNKKHFKEMLATKDITKLDYETQKNIVNNLINKVFVKSGY
IKIEWKIPFKKA

GBS*Int*11.2

MITTNKVAIYVRVSTTNQAEEGYSIEEQKDKLKSYCNIKDWNVFNVYTDGGFSGSNTERPALEQLIKDAKKKKFDTV
LVYKLDRLSRSQKDTLYLIEDIFLENNIDFVSLLENFDTSTPFGKAMVGILSVFAQLEREQIKERMQLGKLGRAKAGKS
MMWAKVAYGYTYHKGSGEMTINELEAIVVREIFNSYLEGMSITKLRDKINDTYPKTPAWSYRIIRQILDNPVYCGYN
QYKGEVYKGNHEPIISEEDFNKTQDELKIRQRTAAEKFNPRPFQAKYMLSGIAQCGYCKAPLKIIMGAVRKDGTRFIK
YECYQRHPRTTRGVTTYNNNQKCHSSSYYKQDVEDYVLREISKLQNDKKAIDELFENTNMDTIDRESIKKQIEAISSKI
KRLNDLYIDDRITIDELRKKSTEFTLSKTFLKEKLENDPILKQQESKDNIKKILSCDDILTMDYDQQKIIVKGLINKVQVT
ADKVIIKWKI

GBS*Int*11.3

MYIEELDDGKYKFIERYIDPLTGKKKRTSVTLDRKTKQAENKARSILQNRISKKINNVTKVELTYGELRQEYLKQWLP
TVKNNTIKNNTRYDEYISYLLDDDVLISNITKATIRNIANELGDKKSYNVVSKCMKRLSAILNYAASLDYIQSNPAKSV
KVIKPVENYDADEKIEFLTIDEMRELYVQMTSKSNKIRDLVVFMFLTGMRYGEVVALTTDKIDFENKTIKINATYDYD
GKELTTPKTENSVRVISVSDSILSIVNDFIVHNRMNNLITDHIFVSRYGNPMSIRYVNKRLKDFMPEKQLKTHVFRHSH
ISYLAEKNVPLKAIMDRVGHKNAETTLKIYTHTTNNMKEYINGQTNINF

GBS*Int*12

MKYTKTKYPNIYFYETAKGKRYYVRRSFFFQGKKKEITKSGLSTIPQARAALTEIERQINEQELGINTQLTVDQYWEIF
SAKRLSTGRWHESSYYLYDSMYRNHIKDEFGFVKLKNLDRNGYEVFIAEKLKKHTRHTVHTINSSFMAILNDAVKNG
NLAGNRLKGVYIGESAIPANNKKITLEQFKEWMDKAKEIMPKKFYALTYMTIFGLRRGEVFGLRPMDVTKNEHGRA
VLKLKDSRSNRTLNGKGSLKTKDSERYVCLDDVGTDYIDYLIDEADRIKRSLGIIKEQKKDYLSINEKGLLINPNQMNK
HFGLVSEAIGIHVTPHMMRHFFTTQSIIAGVPMEQLSQALGHTKIYMTDRYNQVEDELAEATTDMFLTRIR

1.2 Database of amino acid sequences of putative phage inducible-chromosomal islands (PICI) site-specific integrases identified in this study.

PICI1*Int*

MERFMIMKITEVKKKDGTVIYRASIYLGTDKVTGKKVTTKITGRTKKEVREKAKQEAIEFIKNGSTRFKATSITSYQEL
ATLWWDSYKHTVKYNTQLATEKLLTVHVIPIFGAYKLDKLTTPLIQSIINKLADKTNKGERKAYLHYDRIHALNKRIL
QYGVIMQAIPFNPAREVILPRNTKKANTKRVKHFENDELRTFFNYLNNLDKNKYRYFYEVTLYKFLLATGCRINEALA
LNWSDIDLDNAVVHITKTLNYKQEINSPKSKSSYRDIDIDSRTVTMLKQYRRRQIQEAWKLGRSETVVFSDFIHKYPN
NRTLQTRLRTHFKRANVSNIGFHGFRHTHASLLLNTGIPYKELQYRLGHSTLSMTMDIYSHLSKENAKKAVSFFETAIN
SI

PICI2*Int*

MERFMIMKITEVKKKNGATVYRASIYLGVDQVTGKKVKPKVTGRTHKEVKQKANQEKIAFQKDGYTRFKATSIASY
QELSNLWWESYKHTVKPNTQDNVKKLLDNHVIPLFGVYKLDKLTTPLIQSIVNKLADKTNKGEPGAYLHYDKIHALN
KRILQYGVTMQAISSNPARDVVLPRNTQKAKRKKVKHFENQDLKKFLDYLGGLDLSKYRNLYEATLYKFLLATGCR
INEALALSWSDIDLENATISITKTLNHLGQINSPKSKAIYRDIDIDQATITMLKAYQLRQIQEAWKLGRTETVVFSDFIHD

YPNNKTLGTRLKTRFKRAGVPNIGFHGFRHTHASLLLNSGIPYKELQYRLGHSTLSMTMDIYSHLSKENAKKAVSFYE
TALKAL

1.3 Phage inducible-chromosomal islands (PICI) manual detection method

1. Genomes were freshly annotated using Prokka v1.13.1.
2. Manual searches were performed with a text editor for genes annotated as "site-specific integrase", "integrase" or "recombinase".
3. For every genome, after the identification of integrases, manual recognition of the genes at the 5' end and upstream was performed. This was done keeping in mind the basic structure of phage inducible-chromosomal islands (PICI):

   Module A (genes oriented as the integrase)
   - It starts with the **integrase** gene (which could also be annotated as site-specific integrase); there could be a few genes, in variable number and with various functions, oriented in the same direction of the integrase before the next element;
   - The next gene that is always present is named ***stl***; it is a **transcriptional regulator** and it is oriented in the same direction as the integrase (it could be annotated as HTH-domain family protein or DNA-binding protein);

   Module B (genes oriented opposite to the integrase)
   - The next gene that is always present is named ***str***; it is a **transcriptional regulator** and it is oriented in the opposite direction compared to the integrase (it could be annotated as HTH-domain family protein or DNA-binding protein);
   - The number of genes that can be found from here onward is variable and they can have different functions; usually there are a few **replication genes** (e.g. a DNA primase); they are all oriented in the opposite direction compared to the integrase;
   - Sometimes, after the replication genes, genes annotated as **packaging genes** can be present;
   - **Toxin genes** can be found in different parts of the element, usually towards the end.

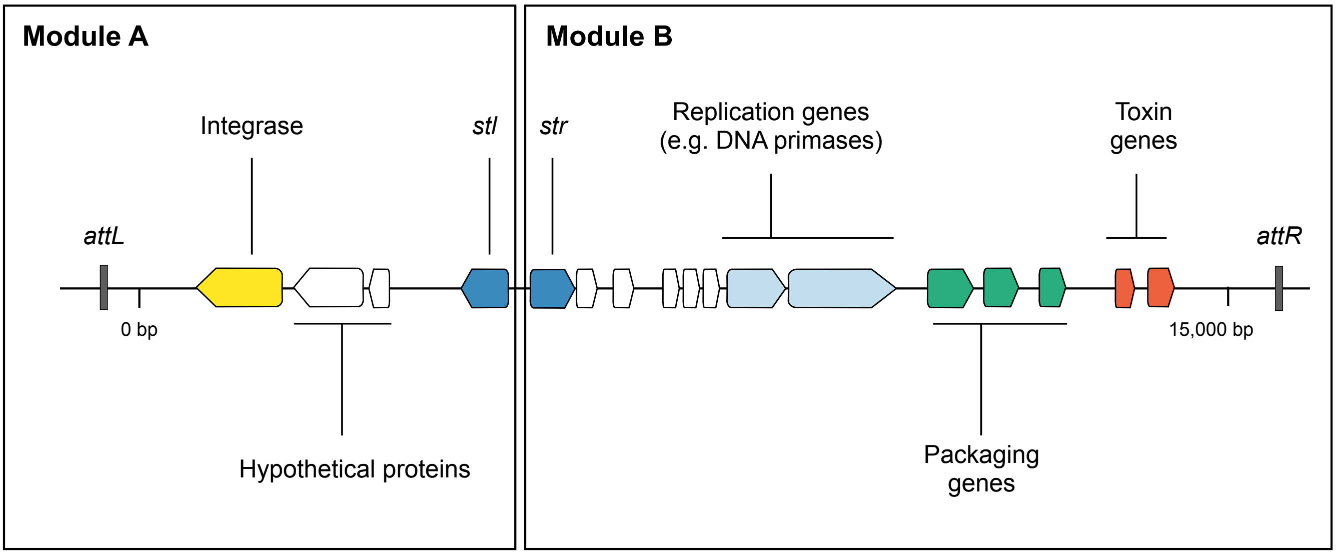

2 SUPPLEMENTARY TABLES AND FIGURES

Table S1: List of 69 Group B *Streptococcus* (GBS) genomes downloaded from NCBI included in dataset 1. Accession numbers, isolate names, host species, country of origin, sequence type (ST) and serotype are shown.

ACCESSION/RUN	ISOLATE	HOST	COUNTRY	ST	SEROTYPE
CP007482.1	138P	fish	USA	ST261	-
CP007565.1	138spar	fish	USA	ST261	-
CP011328.1	GX026	fish	China	ST261	-
CP015976.1	S25	fish	Brazil	ST552	-
CP018623.1	S13	fish	Brazil	ST552	-
CP019800.1	SA30	fish	Brazil	ST552	Ib
CP019801.1	SA33	fish	Brazil	ST552	Ib
CP019802.1	SA53	fish	Brazil	ST260	Ib
CP019803.1	SA73	fish	Brazil	ST260	Ib
CP019804.1	SA1	fish	Brazil	ST552	Ib
CP019805.1	SA5	fish	Brazil	ST552	Ib
CP019806.1	SA9	fish	Brazil	ST552	Ib
CP019807.1	SA16	fish	Brazil	ST552	Ib
CP019808.1	SA75	fish	Brazil	ST260	Ib
CP019809.1	SA79	fish	Brazil	ST552	Ib
CP019810.1	SA81	fish	Brazil	ST552	Ib
CP019811.1	SA85	fish	Brazil	ST927	Ib
CP019812.1	SA95	fish	Brazil	ST927	Ib
CP019813.1	SA97	fish	Brazil	ST927	Ib
CP019814.1	SA102	fish	Brazil	ST927	Ib
CP019815.1	SA132	fish	Brazil	ST260	Ib
CP019816.1	SA136	fish	Brazil	ST260	Ib
CP019817.1	SA159	fish	Brazil	Unknown ST	Ib
CP019818.1	SA184	fish	Brazil	ST552	Ib
CP019819.1	SA191	fish	Brazil	ST260	Ib
CP019820.1	SA195	fish	Brazil	ST552	Ib
CP019821.1	SA201	fish	Brazil	ST552	Ib
CP019822.1	SA209	fish	Brazil	ST552	Ib
CP019823.1	SA212	fish	Brazil	ST552	Ib
CP019824.1	SA218	fish	Brazil	ST927	Ib
CP019825.1	SA220	fish	Brazil	ST552	Ib
CP019826.1	SA245	fish	Brazil	ST260	Ib
CP019827.1	SA256	fish	Brazil	ST260	Ib
CP019828.1	SA289	fish	Brazil	ST260	Ib
CP019829.1	SA330	fish	Brazil	ST552	Ib
CP019830.1	SA333	fish	Brazil	ST552	Ib
CP019831.1	SA341	fish	Brazil	ST552	Ib
CP019832.1	SA343	fish	Brazil	ST552	Ib

Table S1 continued from previous page

ACCESSION/RUN	ISOLATE	HOST	COUNTRY	ST	SEROTYPE
CP019833.1	SA346	fish	Brazil	ST552	Ib
CP019834.1	SA374	fish	Brazil	ST552	Ib
CP019835.1	SA375	fish	Brazil	ST552	Ib
CP019836.1	SA623	fish	Brazil	ST552	Ib
CP019837.1	SA627	fish	Brazil	ST552	Ib
CP025026.1	SGEHI2015-113	fish	Singapore	ST283	-
CP025027.1	SGEHI2015-107	fish	Singapore	ST283	-
CP025028.1	SGEHI2015-95	fish	Singapore	ST283	-
CP025029.1	SGEHI2015-25	fish	Singapore	ST283	-
FO393392.1	2-22	fish	Israel	ST261	Ib
HF952106.1	ILRI112	camel	Kenya	ST617	-
NZ_CP008813.1	C001	bovine	China	ST103	III
NZ_CP012503.1	NGBS357	human	Canada	ST297	-
NZ_CP013908.1	GBS-M002	human	Taiwan	ST1	-
NZ_CP016391.1	FWL1402	frog	China	ST739	-
NZ_CP016501.1	WC1535	fish	China	ST7	-
NZ_CP019978.1	Sag37	human	China	ST12	-
NZ_CP019979.1	Sag158	human	China	ST19	-
NZ_CP020449.1	FDAARGOS_254	-	-	ST22	-
NZ_CP021862.1	CUGBS591	human	Hong Kong	ST12	-
NZ_CP021863.1	SG-M163	human	Singapore	ST283	-
NZ_CP021864.1	SG-M158	human	Singapore	ST283	-
NZ_CP021865.1	SG-M50	human	Singapore	ST283	-
NZ_CP021866.1	SG-M29	human	Singapore	ST283	-
NZ_CP021867.1	SG-M25	human	Singapore	ST19	-
NZ_CP021868.1	SG-M8	human	Singapore	ST1	-
NZ_CP021869.1	SG-M6	human	Singapore	ST17	-
NZ_CP021870.1	SG-M4	human	Singapore	ST23	-
NZ_CP022537.1	874391	human	Japan	ST17	III
NZ_LT545678.1	SA111	bovine	Portugal	ST61	-
NZ_LT714196.1	BM110	human	USA	ST17	-

Table S2: List of 503 Group B *Streptococcus* (GBS) genomes included in dataset 2 (Richards et al., 2019). Accession numbers, isolate names, host species, country of origin, sequence type (ST) with single locus variants (SLV) and serotype are shown.

ACCESSION/RUN	ISOLATE	HOST	COUNTRY	ST	SEROTYPE
AE009948	2603V/R	human	Italy	ST110	V
AEXT0100	FSL_S3-026	bovine	USA	ST67	III
AL732656	NEM316	human	-	ST23	III

Table S2 continued from previous page

ACCESSION/RUN	ISOLATE	HOST	COUNTRY	ST	SEROTYPE
ALQP0100	CCUG_37738	human	Sweden	ST19	III
BCNJ0100	JP17	fish	Thailand	ST283	III
CP000114	A909	human	USA	ST7	Ia
CP003810	GD201008-001	fish	China	ST7	Ia
CP003919	SA20	fish	Brazil	SLV257	Ib
CP006910	CNCTC 10/84	human	USA	ST26	V
CP007570	GBS1-NY	human	USA	ST22	II
CP007571	GBS2-NM	human	USA	ST22	II
CP007572	GBS6	human	USA	ST22	II
CP007631	NGBS061	human	Canada	ST459	IV
CP007632	NGBS572	human	Canada	ST452	IV
CP010319	GBS85147	human	Brazil	ST103	Ia
CP010867	SS1	human	USA	ST1	V
CP010874	CU_GBS_08	human	Hong Kong	ST283	III
CP010875	CU_GBS_98	human	Hong Kong	ST283	III
CP011325	HN016	fish	China	ST7	Ia
CP011326	YM001	fish	China	ST7	Ia
CP011327	GX064	fish	China	ST7	Ia
CP011329	H002	human	China	SLV736	III
CP012419	SG-M1	human	Singapore	ST283	III
CP012480	NGBS128	human	Canada	ST17	III
CP013202	GBS ST1	dog	USA	ST1	V
ERR048526	BE-NI-001	human	Belgium	ST23	Ia
ERR048527	BE-NI-005	human	Belgium	ST8	Ib
ERR048528	BE-NI-007	human	Belgium	ST315	III
ERR048529	BE-NI-008	human	Belgium	ST10	V
ERR048530	DK-NI-001	human	Denmark	ST17	III
ERR048531	DK-NI-002	human	Denmark	ST23	V
ERR048532	DK-NI-003	human	Denmark	ST523	Ib
ERR048534	DK-NI-005	human	Denmark	ST23	Ia
ERR048535	DK-NI-007	human	Denmark	ST17	III
ERR048536	DK-NI-008	human	Denmark	ST9	Ib
ERR048537	DK-NI-009	human	Denmark	ST28	II
ERR048538	DK-NI-010	human	Denmark	ST19	III
ERR048539	DK-NI-011	human	Denmark	ST19	III
ERR048540	DK-NI-012	human	Denmark	SLV1	V
ERR048541	DK-NI-013	human	Denmark	ST1	V
ERR048542	DK-NI-014	human	Denmark	ST88	Ia
ERR048543	DK-NI-015	human	Denmark	ST10	V
ERR048546	DK-NI-021	human	Denmark	ST17	III
ERR048547	DK-NI-022	human	Denmark	SLV1	V
ERR048548	BG-NI-001	human	Bulgaria	ST144	Ia
ERR048549	BG-NI-002	human	Bulgaria	ST17	III

Table S2 continued from previous page

ACCESSION/RUN	ISOLATE	HOST	COUNTRY	ST	SEROTYPE
ERR048550	BG-NI-003	human	Bulgaria	ST8	Ib
ERR048551	BG-NI-004	human	Bulgaria	ST23	Ia
ERR048552	BG-NI-005	human	Bulgaria	ST23	Ia
ERR048553	BG-NI-006	human	Bulgaria	ST12	Ib
ERR048554	BG-NI-007	human	Bulgaria	ST12	Ib
ERR048555	BG-NI-009	human	Bulgaria	ST28	II
ERR048556	BG-NI-010	human	Bulgaria	ST12	II
ERR048557	BG-NI-011	human	Bulgaria	SLV1	V
ERR048561	DE-NI-001	human	Germany	SLV1	V
ERR048562	DE-NI-003	human	Germany	ST10	V
ERR048563	DE-NI-004	human	Germany	ST10	V
ERR048564	DE-NI-006	human	Germany	ST144	Ia
ERR048567	DE-NI-012	human	Germany	ST88	Ia
ERR048568	DE-NI-0013	human	Germany	ST17	III
ERR048569	DE-NI-014	human	Germany	ST23	Ia
ERR048570	DE-NI-0017	human	Germany	SLV17	III
ERR048571	DE-NI-0019	human	Germany	ST17	III
ERR048572	DE-NI-022	human	Germany	ST387	V
ERR048573	DE-NI-032	human	Germany	ST17	III
ERR048574	DE-NI-033	human	Germany	ST23	Ia
ERR048575	DE-NI-036	human	Germany	SLV17	III
ERR048576	DE-NI-037	human	Germany	ST17	III
ERR048577	DE-NI-040	human	Germany	ST23	Ia
ERR048579	DE-NI-042	human	Germany	ST19	III
ERR048581	IT-NI-007	human	Italy	ST17	III
ERR048582	IT-NI-008	human	Italy	ST17	III
ERR048583	IT-NI-009	human	Italy	ST17	III
ERR048584	IT-NI-016	human	Italy	ST130	IX
ERR048586	IT-NI-019	human	Italy	ST467	III
ERR048587	IT-NI-020	human	Italy	SLV17	III
ERR048588	IT-NI-028	human	Italy	ST1	V
ERR048589	IT-NI-0031	human	Italy	SLV1	V
ERR048591	IT-NI-033	human	Italy	ST17	III
ERR048592	IT-NI-034	human	Italy	ST17	III
ERR048594	IT-NI-037	human	Italy	ST26	V
ERR048595	CZ-NI-001	human	Czech Republic	ST23	Ia
ERR048596	CZ-NI-002	human	Czech Republic	ST19	III
ERR048597	CZ-NI-003	human	Czech Republic	ST19	III
ERR048598	CZ-NI-004	human	Czech Republic	SLV1	V
ERR048599	CZ-NI-005	human	Czech Republic	ST23	Ia
ERR048600	CZ-NI-006	human	Czech Republic	ST1	V
ERR048601	CZ-NI-007	human	Czech Republic	ST255	Ib
ERR048602	CZ-NI-008	human	Czech Republic	ST1	V

Table S2 continued from previous page

ACCESSION/RUN	ISOLATE	HOST	COUNTRY	ST	SEROTYPE
ERR048603	CZ-NI-009	human	Czech Republic	ST1	V
ERR048605	CZ-NI-013	human	Czech Republic	ST1	V
ERR048606	CZ-NI-014	human	Czech Republic	ST479	II
ERR048607	CZ-NI-015	human	Czech Republic	ST1	V
ERR048608	CZ-NI-016	human	Czech Republic	ST459	IV
ERR048611	GB-NI-003	human	United Kingdom	ST17	III
ERR048612	GB-NI-004	human	United Kingdom	ST17	III
ERR048613	GB-NI-005	human	United Kingdom	SLV17	III
ERR048614	GB-NI-006	human	United Kingdom	ST23	Ia
ERR048615	GB-NI-007	human	United Kingdom	ST19	III
ERR048616	GB-NI-009	human	United Kingdom	ST1	V
ERR048617	GB-NI-010	human	United Kingdom	ST1	V
ERR048618	GB-NI-011	human	United Kingdom	ST19	III
ERR054970	B09PS	human	Australia	ST1	V
ERR054971	B15VD	human	Australia	ST17	III
ERR054972	B24VD	human	Australia	ST28	II
ERR054973	B37VS	human	Australia	ST335	III
ERR054974	B42VD	human	Australia	ST23	III
ERR054975	B50VD	human	Australia	ST23	Ia
ERR054976	B68VD	human	Australia	SLV19	III
ERR054982	RBH02	human	Australia	ST2	IV
ERR054983	RBH03	human	Australia	ST19	III
ERR054985	RBH05	human	Australia	ST1	V
ERR054987	RBH07	human	Australia	ST23	Ia
ERR054988	RBH08	human	Australia	ST23	Ia
ERR054990	RBH11	human	Australia	ST19	III
ERR054992	B96P	human	Australia	ST17	III
ERR054993	B41VS	human	Australia	ST652	II
ERR054994	B50VS	human	Australia	ST23	Ia
ERR054997	B96V	human	Australia	ST17	III
ERR829829	MRI Z1-116	bovine	Denmark	ST604	Ib
ERR829883	MRI Z2-084	human	Finland	ST28	II
HF952104	09mas018883	bovine	Sweden	ST1	V
HF952105	ILRI005	camel	Kenya	ST609	V
HG939456	COH1	human	USA	ST17	III
JPOV0100	NGS-ED-1000	human	United Kingdom	ST7	Ia
SRR1213207	NGBS024	human	Canada	ST459	IV
SRR1213208	NGBS046	human	Canada	ST459	IV
SRR1213210	NGBS058	human	Canada	ST459	IV
SRR1213213	NGBS070	human	Canada	ST459	IV
SRR1213214	NGBS100	human	Canada	ST452	IV
SRR1213215	NGBS122	human	Canada	ST452	IV
SRR1213216	NGBS146	human	Canada	ST459	IV

Table S2 continued from previous page

ACCESSION/RUN	ISOLATE	HOST	COUNTRY	ST	SEROTYPE
SRR1213217	NGBS151	human	Canada	ST3	IV
SRR1213218	NGBS187	human	Canada	ST452	IV
SRR1213219	NGBS191	human	Canada	ST459	IV
SRR1213220	NGBS197	human	Canada	ST452	IV
SRR1213221	NGBS199	human	Canada	ST459	IV
SRR1213223	NGBS290	human	Canada	ST459	IV
SRR1213224	NGBS314	human	Canada	ST452	IV
SRR1213226	NGBS379	human	Canada	ST3	IV
SRR1213227	NGBS400	human	Canada	SLV459	IV
SRR1213228	NGBS410	human	Canada	ST459	IV
SRR1213229	NGBS447	human	Canada	ST196	IV
SRR1213230	NGBS472	human	Canada	SLV196	IV
SRR1213231	NGBS493	human	Canada	ST459	IV
SRR1213232	NGBS507	human	Canada	ST459	IV
SRR1213233	NGBS521	human	Canada	ST459	IV
SRR1213234	NGBS525	human	Canada	ST459	IV
SRR1213235	NGBS528	human	Canada	ST459	IV
SRR1213236	NGBS556	human	Canada	ST452	IV
SRR1213238	NGBS572	human	Canada	ST452	IV
SRR1213239	NGBS588	human	Canada	ST682	IV
SRR1213240	NGBS597	human	Canada	ST452	IV
SRR1213241	NGBS598	human	Canada	ST452	IV
SRR1213242	NGBS612	human	Canada	ST452	IV
SRR1213243	NGBS615	human	Canada	ST459	IV
SRR1790740	NGBS010	human	Canada	ST1	V
SRR1790741	NGBS107	human	Canada	ST1	V
SRR1790742	NGBS110	human	Canada	ST1	V
SRR1790743	NGBS117	human	Canada	ST1	V
SRR1790749	NGBS180	human	Canada	ST1	V
SRR1790751	NGBS021	human	Canada	ST1	V
SRR1790752	NGBS210	human	Canada	ST1	V
SRR1790753	NGBS022	human	Canada	ST1	V
SRR1790758	NGBS246	human	Canada	ST1	V
SRR1790759	NGBS025	human	Canada	ST1	V
SRR1790760	NGBS267	human	Canada	ST1	V
SRR1790761	NGBS272	human	Canada	ST1	V
SRR1790765	NGBS028	human	Canada	ST1	V
SRR1790766	NGBS283	human	Canada	ST1	V
SRR1790767	NGBS287	human	Canada	ST1	V
SRR1790768	NGBS288	human	Canada	ST1	V
SRR1790769	NGBS298	human	Canada	ST1	V
SRR1790770	NGBS030	human	Canada	ST1	V
SRR1790771	NGBS303	human	Canada	ST1	V

Table S2 continued from previous page

ACCESSION/RUN	ISOLATE	HOST	COUNTRY	ST	SEROTYPE
SRR1790773	NGBS323	human	Canada	ST1	V
SRR1790775	NGBS330	human	Canada	ST1	V
SRR1790779	NGBS348	human	Canada	ST1	V
SRR1790780	NGBS035	human	Canada	ST1	V
SRR1790782	NGBS359	human	Canada	ST1	V
SRR1790783	NGBS360	human	Canada	ST1	V
SRR1790785	NGBS380	human	Canada	ST1	V
SRR1790786	NGBS381	human	Canada	ST1	V
SRR1790788	NGBS418	human	Canada	ST1	V
SRR1790789	NGBS425	human	Canada	ST1	V
SRR1790790	NGBS434	human	Canada	ST1	V
SRR1790792	NGBS444	human	Canada	ST1	V
SRR1790793	NGBS462	human	Canada	ST1	V
SRR1790794	NGBS492	human	Canada	ST1	V
SRR1790795	NGBS494	human	Canada	ST1	V
SRR1790796	NGBS497	human	Canada	ST1	V
SRR1790797	NGBS499	human	Canada	ST1	V
SRR1790799	NGBS519	human	Canada	ST1	V
SRR1790800	NGBS536	human	Canada	ST1	V
SRR1790801	NGBS054	human	Canada	ST1	V
SRR1790802	NGBS553	human	Canada	ST1	V
SRR1790803	NGBS558	human	Canada	ST1	V
SRR1790805	NGBS571	human	Canada	SLV1	V
SRR1790806	NGBS579	human	Canada	ST1	V
SRR1790807	NGBS580	human	Canada	ST1	V
SRR1790808	NGBS586	human	Canada	ST1	V
SRR1790809	NGBS604	human	Canada	ST1	V
SRR1790811	NGBS063	human	Canada	ST1	V
SRR1790812	NGBS630	human	Canada	ST1	V
SRR1790814	NGBS068	human	Canada	ST1	V
SRR1790815	NGBS008	human	Canada	ST1	V
SRR1790816	NGBS009	human	Canada	ST1	V
SRR1790817	NGBS092	human	Canada	ST1	V
SRR1790819	NGBS094	human	Canada	ST1	V
SRR1790820	NGBS099	human	Canada	ST1	V
SRR2062051	NGBS680	human	Canada	ST459	IV
SRR2062052	NGBS686	human	Canada	ST459	IV
SRR2062054	NGBS762	human	Canada	ST459	IV
SRR2062055	NGBS767	human	Canada	ST459	IV
SRR2062056	NGBS768	human	Canada	ST452	IV
SRR2062057	NGBS783	human	Canada	ST459	IV
SRR2062058	NGBS788	human	Canada	ST459	IV
SRR2062059	NGBS789	human	Canada	ST459	IV

Table S2 continued from previous page

ACCESSION/RUN	ISOLATE	HOST	COUNTRY	ST	SEROTYPE
SRR2062060	NGBS791	human	Canada	ST459	IV
SRR2062063	NGBS795	human	Canada	ST459	IV
SRR2062064	NGBS798	human	Canada	ST710	IV
SRR2062065	NGBS800	human	Canada	ST459	IV
SRR2062066	NGBS698e	human	Canada	ST459	IV
SRR2062068	NGBS801	human	Canada	ST459	IV
SRR2062069	NGBS806	human	Canada	ST459	IV
SRR2062071	NGBS808	human	Canada	ST459	IV
SRR2062072	NGBS809	human	Canada	ST459	IV
SRR2062074	NGBS813	human	Canada	ST459	IV
SRR2062075	NGBS815	human	Canada	ST459	IV
SRR2062076	NGBS824	human	Canada	ST711	IV
SRR2062077	NGBS825	human	Canada	ST459	IV
SRR2062079	NGBS830	human	Canada	ST459	IV
SRR2062080	NGBS836	human	Canada	ST459	IV
SRR2062081	NGBS700e	human	Canada	ST459	IV
SRR2062082	NGBS855	human	Canada	ST459	IV
SRR2062084	NGBS860	human	Canada	ST459	IV
SRR2062085	NGBS877	human	Canada	SLV459	IV
SRR2062086	NGBS899	human	Canada	SLV459	IV
SRR2062087	NGBS904	human	Canada	ST459	IV
SRR2062088	NGBS933	human	Canada	ST3	IV
SRR2062090	NGBS956	human	Canada	ST459	IV
SRR2062091	NGBS960	human	Canada	ST3	IV
SRR2062092	NGBS964	human	Canada	ST459	IV
SRR2062093	NGBS965	human	Canada	ST459	IV
SRR2062094	NGBS702	human	Canada	ST459	IV
SRR2062097	NGBS977f	human	Canada	SLV459	IV
SRR2062099	NGBS979	human	Canada	ST459	IV
SRR2062100	NGBS984	human	Canada	ST459	IV
SRR2062101	NGBS991	human	Canada	ST196	IV
SRR2062103	NGBS996	human	Canada	ST459	IV
SRR2062104	NGBS1006	human	Canada	ST459	IV
SRR2062105	NGBS1009	human	Canada	ST459	IV
SRR2062107	NGBS1017	human	Canada	ST459	IV
SRR2062109	NGBS706	human	Canada	ST459	IV
SRR2062110	NGBS1021	human	Canada	ST459	IV
SRR2062112	NGBS1024	human	Canada	ST459	IV
SRR2062125	NGBS727	human	Canada	SLV452	IV
SRR2062139	NGBS736	human	Canada	ST459	IV
SRR2062154	NGBS737	human	Canada	ST459	IV
SRR2062160	NGBS741	human	Canada	ST459	IV
SRR2068019	NGBS1041	human	Canada	ST459	IV

Table S2 continued from previous page

ACCESSION/RUN	ISOLATE	HOST	COUNTRY	ST	SEROTYPE
SRR2068021	NGBS1043	human	Canada	ST459	IV
SRR2068022	NGBS1045	human	Canada	ST459	IV
SRR2068023	NGBS1046	human	Canada	ST459	IV
SRR2068024	NGBS1047	human	Canada	ST459	IV
SRR2068025	NGBS1048	human	Canada	ST459	IV
SRR2068026	NGBS1049	human	Canada	ST459	IV
SRR2068027	NGBS1050	human	Canada	ST452	IV
SRR2068028	NGBS1051	human	Canada	ST459	IV
SRR2068029	NGBS1052	human	Canada	ST459	IV
SRR2068031	NGBS1054	human	Canada	ST459	IV
SRR2068032	NGBS1056	human	Canada	ST459	IV
SRR2068033	NGBS1058	human	Canada	ST459	IV
SRR2068034	NGBS1059	human	Canada	ST459	IV
SRR2068035	NGBS1061	human	Canada	ST459	IV
SRR2068036	NGBS1062	human	Canada	ST459	IV
SRR2068037	NGBS1063	human	Canada	ST459	IV
SRR2068038	NGBS1064	human	Canada	ST459	IV
SRR2068039	NGBS1065	human	Canada	ST459	IV
SRR2068040	NGBS1066	human	Canada	ST459	IV
SRR2068041	NGBS1067	human	Canada	ST459	IV
SRR2068042	NGBS1068	human	Canada	ST459	IV
SRR2068043	NGBS1071g	human	Canada	ST459	IV
SRR2068044	NGBS1072g	human	Canada	ST459	IV
SRR2068045	NGBS1074	human	Canada	ST459	IV
SRR2068046	NGBS1075	human	Canada	ST459	IV
SRR2068047	NGBS1079	human	Canada	ST452	IV
SRR2068048	NGBS1080	human	Canada	SLV459	IV
SRR2068049	NGBS1082	human	Canada	ST459	IV
SRR2068050	NGBS1083	human	Canada	ST459	IV
SRR2451885	NGBS129	human	Canada	ST31	III
SRR2451888	NGBS147	human	Canada	ST17	III
SRR2451889	NGBS149	human	Canada	ST17	III
SRR2451892	NGBS169	human	Canada	ST17	III
SRR2451894	NGBS205	human	Canada	ST17	III
SRR2451896	NGBS222	human	Canada	ST17	III
SRR2451897	NGBS238	human	Canada	ST17	III
SRR2451898	NGBS239	human	Canada	ST17	III
SRR2451901	NGBS277	human	Canada	ST17	III
SRR2451902	NGBS282	human	Canada	ST17	III
SRR2451904	NGBS296	human	Canada	ST17	III
SRR2451905	NGBS297	human	Canada	ST17	III
SRR2451906	NGBS299	human	Canada	ST17	III
SRR2451907	NGBS306	human	Canada	ST17	III

Table S2 continued from previous page

ACCESSION/RUN	ISOLATE	HOST	COUNTRY	ST	SEROTYPE
SRR2451908	NGBS312	human	Canada	ST17	III
SRR2451909	NGBS317	human	Canada	ST17	III
SRR2451910	NGBS318	human	Canada	ST290	III
SRR2451914	NGBS356	human	Canada	ST17	III
SRR2451915	NGBS361	human	Canada	ST17	III
SRR2451916	NGBS362	human	Canada	ST17	III
SRR2451917	NGBS368	human	Canada	ST17	III
SRR2451919	NGBS374	human	Canada	ST17	III
SRR2451922	NGBS398	human	Canada	ST17	III
SRR2451923	NGBS403	human	Canada	ST17	III
SRR2451925	NGBS421	human	Canada	ST17	III
SRR2451926	NGBS422	human	Canada	ST17	III
SRR2451929	NGBS456	human	Canada	ST17	III
SRR2451930	NGBS464	human	Canada	ST17	III
SRR2451931	NGBS469	human	Canada	ST17	III
SRR2451932	NGBS470	human	Canada	ST17	III
SRR2451933	NGBS483	human	Canada	ST17	III
SRR2451934	NGBS485	human	Canada	ST17	III
SRR2451935	NGBS486	human	Canada	ST17	III
SRR2451936	NGBS500	human	Canada	ST17	III
SRR2451938	NGBS502	human	Canada	ST95	III
SRR2451939	NGBS515	human	Canada	ST17	III
SRR2451942	NGBS534	human	Canada	ST17	III
SRR2451943	NGBS551	human	Canada	ST17	III
SRR2451945	NGBS583	human	Canada	ST17	III
SRR2451946	NGBS593	human	Canada	ST17	III
SRR2451947	NGBS594	human	Canada	ST17	III
SRR2451948	NGBS596	human	Canada	ST17	III
SRR2451949	NGBS607	human	Canada	ST17	III
SRR2451950	NGBS608	human	Canada	ST17	III
SRR2451951	NGBS609	human	Canada	ST17	III
SRR2451952	NGBS613	human	Canada	ST17	III
SRR2451954	NGBS618	human	Canada	ST17	III
SRR2451955	NGBS622	human	Canada	ST148	III
SRR2451958	NGBS632	human	Canada	ST17	III
SRR2451960	NGBS641	human	Canada	ST17	III
SRR2451961	NGBS644	human	Canada	ST17	III
SRR2451962	NGBS650	human	Canada	ST17	III
SRR2981533	SGBS103	human	USA	ST1	V
SRR2981534	SGBS104	human	USA	ST1	V
SRR2981535	SGBS105	human	USA	SLV1	V
SRR2981536	SGBS106	human	USA	ST1	V
SRR2981541	SGBS111	human	USA	ST1	V

Table S2 continued from previous page

ACCESSION/RUN	ISOLATE	HOST	COUNTRY	ST	SEROTYPE
SRR2981542	SGBS114	human	USA	ST1	V
SRR2981543	SGBS115	human	USA	ST1	V
SRR2981545	SGBS118	human	USA	ST1	V
SRR2981546	SGBS119	human	USA	ST1	V
SRR2981547	SGBS120	human	USA	ST1	V
SRR2981548	SGBS122	human	USA	ST1	V
SRR2981550	SGBS126	human	USA	ST1	V
SRR2981554	SGBS133	human	USA	ST1	V
SRR2981555	SGBS135	human	USA	ST1	V
SRR2981558	SGBS140	human	USA	ST1	V
SRR2981559	SGBS141	human	USA	ST1	V
SRR2981560	SGBS143	human	USA	ST1	V
SRR2981561	SGBS144	human	USA	ST1	V
SRR2981562	SGBS145	human	USA	ST1	V
SRR2981563	SGBS146	human	USA	ST1	V
SRR2981564	SGBS147	human	USA	ST1	V
SRR2981565	SGBS148	human	USA	ST1	V
SRR2981566	SGBS150	human	USA	ST1	V
SRR2981568	SGBS152	human	USA	ST1	V
SRR2981569	SGBS031	human	USA	ST1	V
SRR2981570	SGBS032	human	USA	ST1	V
SRR2981571	SGBS033	human	USA	ST1	V
SRR2981572	SGBS034	human	USA	ST1	V
SRR2981573	SGBS035	human	USA	ST1	V
SRR2981574	SGBS036	human	USA	ST1	V
SRR2981575	SGBS037	human	USA	ST1	V
SRR2981576	SGBS038	human	USA	ST1	V
SRR2981577	SGBS039	human	USA	ST1	V
SRR2981578	SGBS040	human	USA	ST1	V
SRR2981579	SGBS041	human	USA	ST1	V
SRR2981580	SGBS042	human	USA	ST1	V
SRR2981582	SGBS044	human	USA	ST1	V
SRR2981583	SGBS045	human	USA	ST1	V
SRR2981584	SGBS046	human	USA	ST1	V
SRR2981585	SGBS047	human	USA	ST1	V
SRR2981586	SGBS048	human	USA	ST1	V
SRR2981587	SGBS049	human	USA	SLV1	V
SRR2981589	SGBS051	human	USA	ST1	V
SRR2981590	SGBS052	human	USA	ST1	V
SRR2981591	SGBS053	human	USA	ST1	V
SRR2981592	SGBS054	human	USA	ST1	V
SRR2981593	SGBS056	human	USA	ST1	V
SRR2981594	SGBS057	human	USA	ST1	V

Table S2 continued from previous page

ACCESSION/RUN	ISOLATE	HOST	COUNTRY	ST	SEROTYPE
SRR2981595	SGBS058	human	USA	ST1	V
SRR2981597	SGBS060	human	USA	ST1	V
SRR2981604	SGBS067	human	USA	ST1	V
SRR2981606	SGBS069	human	USA	ST1	V
SRR2981610	SGBS074	human	USA	ST1	V
SRR2981611	SGBS075	human	USA	ST1	V
SRR2981612	SGBS076	human	USA	ST1	V
SRR2981613	SGBS077	human	USA	ST1	V
SRR2981614	SGBS078	human	USA	ST1	V
SRR2981615	SGBS079	human	USA	ST153	V
SRR2981618	SGBS082	human	USA	ST1	V
SRR2981620	SGBS084	human	USA	ST1	V
SRR2981621	SGBS085	human	USA	ST1	V
SRR2981623	SGBS087	human	USA	ST1	V
SRR2981626	SGBS092	human	USA	ST1	V
SRR2981628	SGBS094	human	USA	ST1	V
SRR2981630	SGBS096	human	USA	ST1	V
SRR2981632	SGBS098	human	USA	ST1	V
SRR494266	CCUG_24810	human	Sweden	ST19	III
SRR494270	CCUG_37430	human	Sweden	ST19	II
SRR494271	CCUG_29376	human	Sweden	ST12	Ib
SRR494272	CCUG_30636	human	Sweden	ST1	V
SRR494276	FSL_S3-137	human	USA	ST8	Ib
SRR494279	FSL_S3-001	human	USA	ST1	V
SRR494280	FSL_S3-003	human	USA	ST19	III
SRR494281	CCUG_91	human	Sweden	ST28	II
SRR494284	FSL_S3-102	human	USA	ST31	III
SRR494285	FSL_F2-343	human	USA	ST88	Ia
SRR494286	FSL_S3-014	human	USA	ST8	Ib
SRR494288	FSL_S3-090	human	USA	ST23	Ia
SRR494289	FSL_S3-023	human	USA	ST1	V
SRR494292	LMG_15085	human	USA	ST17	III
SRR494295	CCUG_49086	human	Sweden	ST17	III
SRR494296	CCUG_49100	human	Sweden	ST1	V
SRR494297	CCUG_44140	human	Sweden	ST1	V
SRR494298	LMG_15081	human	USA	ST25	Ia
SRR494299	LMG_15083	human	USA	ST7	Ia
SRR494300	LMG_15084	human	USA	ST19	II
SRR494302	CCUG_49072	human	Sweden	ST524	V
SRR494303	CCUG_49087	human	Sweden	ST17	III
SRR494306	CCUG_47293	human	Sweden	SLV9	Ib
SRR494309	CCUG_44074	human	Sweden	ST23	Ia
SRR494311	CCUG_39096_A	human	Sweden	ST9	Ib

Table S2 continued from previous page

ACCESSION/RUN	ISOLATE	HOST	COUNTRY	ST	SEROTYPE
SRR494317	CCUG_37739	human	Sweden	ST23	Ia
SRR494322	BSU253	human	Germany	ST23	Ia
SRR494323	BSU247	human	Germany	ST26	V
SRR494325	BSU248	human	Germany	ST12	Ib
SRR494327	BSU252	human	Germany	ST1	V
SRR494328	BSU454	human	Germany	ST8	Ib
SRR494330	LMG_15094	human	Belgium	ST17	III
SRR494331	LMG_15095	human	Belgium	ST17	III
SRR494332	LMG_15090	human	Belgium	ST8	Ib
SRR494336	LMG_15091	human	Belgium	SLV786	IV
SRR494339	BSU451	human	Germany	ST103	Ia
SRR494340	BSU96	human	Germany	ST17	III
SRR494341	BSU165	human	Germany	ST28	II
SRR494342	BSU174	human	Germany	ST41	V
SRR494343	BSU92	human	Germany	ST196	IV
SRR494344	BSU133	human	Germany	ST6	Ib
SRR494346	BSU260	human	Germany	ST88	Ia
SRR494355	GB00202	human	Canada	ST10	Ib
SRR494358	GB00097	human	Canada	ST17	III
SRR494359	GB00111	human	Canada	ST32	III
SRR494360	GB00115	human	Canada	ST17	III
SRR494361	GB00190	human	Canada	ST23	Ia
SRR494364	GB00083	human	Canada	ST1	VI
SRR494365	GB00084	human	Canada	ST1	VIII
SRR494366	GB00003	human	Canada	ST12	Ib
SRR494367	GB00012	human	Canada	ST1	V
SRR494368	GB00018	human	Canada	ST444	Ia
SRR494369	GB00082	human	Canada	ST2	IV
SRR494370	GB00013	human	Canada	ST1	V
SRR494371	GB00020	human	Canada	ST1	V
SRR494372	GB00002	human	Canada	ST23	Ia
SRR494374	GB00864	human	USA	ST10	II
SRR494375	GB00663	human	Canada	ST19	III
SRR494376	GB00679	human	Canada	ST2	II
SRR494377	GB00654	human	Canada	ST17	III
SRR494378	GB00651	human	Canada	ST8	Ib
SRR494380	GB00640	human	Canada	ST26	V
SRR494383	GB00588	human	Canada	ST447	II
SRR494386	GB00555	human	Canada	ST12	Ib
SRR494388	GB00264	human	Canada	ST10	II
SRR494389	GB00279	human	Canada	ST2	II
SRR494390	GB00300	human	Canada	ST130	IX
SRR494392	GB00241	human	Canada	ST1	V

Table S2 continued from previous page

ACCESSION/RUN	ISOLATE	HOST	COUNTRY	ST	SEROTYPE
SRR494393	GB00226	human	Canada	ST28	II
SRR494394	GB00245	human	Canada	ST23	Ia
SRR494395	GB00247	human	Canada	ST24	Ia
SRR494396	GB00219	human	Canada	ST8	Ib
SRR494568	GB00932	human	USA	ST23	Ia
SRR494611	GB00887	human	USA	ST23	Ia
SRR494612	GB00888	human	USA	ST41	V
SRR494631	GB00914	human	USA	ST8	Ib
SRR494632	GB00922	human	USA	ST88	Ia
SRR494635	GB00911	human	USA	ST452	IV
SRR494636	GB00867	human	USA	ST23	Ia
SRR494637	GB00884	human	USA	ST19	III
SRR494638	GB00955	human	USA	SLV1	V
SRR494645	GB00924	human	USA	ST1	V
SRR494656	GB00933	human	USA	ST452	IV
SRR494658	GB00901	human	USA	ST459	IV
SRR494659	GB00909	human	USA	ST12	Ib
SRR494660	GB00874	human	USA	ST1	II
SRR496544	MRI_Z1-211	bovine	Italy	ST1	V
SRR496556	MRI_Z1-025	bovine	Denmark	ST1	V
SRR496920	MRI_Z1-199	seal	United Kingdom	ST23	Ia
SRR497007	GB00893	human	USA	ST8	Ib
SRR497011	STIR-CD-14	fish	Vietnam	ST491	III
SRR497118	MRI_Z1-198	dolphin	United Kingdom	ST12	Ib
SRR525043	GB00548	human	Canada	ST88	Ia
SRR628712	GB00999	human	USA	ST1	V
SRR6996453	QMA0323	fish	Australia	ST261	Ib

Table S3: Clonal complexes (CC) of the genomes included in dataset 2. Sequence types (ST) and single locus variants (SLV) identified and grouped among the different CC according to Richards et al. (2019) are shown.

CLONAL COMPLEX (CC)	SEQUENCE TYPES (ST)
**1**	1, 2, 153, 196, 387, 459, 524, 682, 710
**7**	6, 7, 41
**12**	3, 8, 9, 10, 12, 255, 523, 604, 652, 711
**17**	17, 31, 32, 95, 148, 290, 315, 467
**19**	19, 28, 110, 335, 447, 479
**22**	22
**23**	23, 24, 25, 88, 144, 452
**26**	26
**67**	67
**103**	103
**130**	130
**260/261**	261, 257 (SLV)
**283**	283, 491
**609**	609

Table S4. Prophage integrase family type identity and similarity table. Progressive numbers are assigned to sixteen integrase types, at twelve different integration sites. Numbers indicate query coverage and percentage of identity (QC/ID) based on BLASTp comparison of the amino acid sequences. Comparison was always run with the longest sequence as the database and the shortest sequence as the query. Phage integrase 1 was present at insertion site GBS1 and at GBS7 (1at7).

PHAGE INTEGRASE	1	2.1	2.2	3	4	5	6.1	6.2	1at7	8	9.1	9.2	10	11.1	11.2	11.3	12
**1**	100/ 100	84/ 23.68	87/ 25.15	92/ 26.69	91/ 28.14	51/ 26.71	97/ 35.7	98/ 33.16	100/ 100	99/ 28.72	92/ 24.35	93/ 22.25	18/ 32.26	38/ 24.24	4/ 27.78	92/ 26.76	39/ 36.84
**2.1**	84/ 23.68	100/ 100	100/ 71.35	49/ 28.10	45/ 24.04	67/ 21.61	94/ 26.86	96/ 27.73	84/ 23.68	80/ 28.99	99/ 59.32	99/ 58.92	17/ 50	25/ 40.74	34/ 25.58	78/ 24.23	90/ 25.14
**2.2**	87/ 25.15	100/ 71.35	100/ 100	42/ 30.46	87/ 20.30	45/ 24.14	89/ 23.35	93/ 26.29	87/ 25.15	76/ 23.67	99/ 54.65	99/ 54.26	13/ 45.45	15/ 34.78	5/ 57.14	77/ 27.21	86/ 22.69
**3**	92/ 26.69	49/ 28.10	42/ 30.46	100/ 100	95/ 31.75	58/ 23.70	94/ 29.84	89/ 26.11	92/ 26.69	97/ 34.75	78/ 26.48	81/ 23.97	29/ 26.80	21/ 28.26	17/ 26.32	98/ 50.83	90/ 19.83
**4**	91/ 28.14	45/ 24.04	87/ 20.30	95/ 31.75	100/ 100	91/ 19.49	80/ 27.30	93/ 28.12	91/ 28.14	97/ 34.04	39/ 22.70	85/ 22.09	17/ 32.56	31/ 35.29	0/ 0	97/ 32.55	39/ 24.07
**5**	51/ 26.71	67/ 21.61	45/ 24.14	58/ 23.70	91/ 19.49	100/ 100	59/ 30.16	72/ 24.31	51/ 26.71	77/ 23.08	46/ 25.77	74/ 19.76	15/ 50.00	53/ 30.00	20/ 25.93	72/ 24.70	22/ 42.86
**6.1**	97/ 35.7	94/ 26.86	89/ 23.35	94/ 29.84	80/ 27.30	59/ 30.16	100/ 100	98/ 58.06	97/ 35.7	81/ 28.16	94/ 27.20	93/ 26.15	17/ 47.06	45/ 33.33	30/ 17.74	90/ 29.18	90/ 22.75
**6.2**	98/ 33.16	96/ 27.73	93/ 26.29	89/ 26.11	93/ 28.12	72/ 24.31	98/ 58.06	100/ 100	98/ 33.16	82/ 27.85	97/ 27.12	96/ 26.57	43/ 29.82	11/ 50.00	30/ 32.43	95/ 28.61	91/ 23.99
**1at7**	100/ 100	84/ 23.68	87/ 25.15	92/ 26.69	91/ 28.14	51/ 26.71	97/ 35.7	98/ 33.16	100/ 100	99/ 28.72	92/ 24.35	93/ 22.25	18/ 32.26	38/ 24.24	4/ 27.78	92/ 26.76	39/ 36.84
**8**	99/ 28.72	80/ 28.99	76/ 23.67	97/ 34.75	97/ 34.04	77/ 23.08	81/ 28.16	82/ 27.85	99/ 28.72	100/ 100	83/ 23.30	93/ 23.03	36/ 36.84	34/ 35.14	3/ 36.84	96/ 36.54	72/ 23.10
**9.1**	92/ 24.35	99/ 59.32	99/ 54.65	78/ 26.48	39/ 22.70	46/ 25.77	94/ 27.20	97/ 27.12	92/ 24.35	83/ 23.30	100/ 100	100/ 83.71	13/ 38.46	20/ 45.16	16/ 25.00	78/ 23.45	84/ 23.19
**9.2**	93/ 22.25	99/ 58.92	99/ 54.26	81/ 23.97	85/ 22.09	74/ 19.76	93/ 26.15	96/ 26.57	93/ 22.25	93/ 23.03	100/ 83.71	100/ 100	12/ 39.13	19/ 40.74	18/ 25.00	90/ 24.49	85/ 22.75
**10**	18/ 32.26	17/ 50	13/ 45.45	29/ 26.80	17/ 32.56	15/ 50.00	17/ 47.06	43/ 29.82	18/ 32.26	36/ 36.84	13/ 38.46	12/ 39.13	100/ 100	99/ 44.54	98/ 47.16	37/ 36.84	32/ 30.95
**11.1**	38/ 24.24	25/ 40.74	15/ 34.78	21/ 28.26	31/ 35.29	53/ 30.00	45/ 33.33	11/ 50.00	38/ 24.24	34/ 35.14	20/ 45.16	19/ 40.74	99/ 44.54	100/ 100	99/ 57.92	16/ 27.27	28/ 23.08
**11.2**	4/ 27.78	34/ 25.58	5/ 57.14	17/ 26.32	0/ 0	20/ 25.93	30/ 17.74	30/ 32.43	4/ 27.78	3/ 36.84	16/ 25.00	18/ 25.00	98/ 47.16	99/ 57.92	100/ 100	7/ 42.86	2/ 54.55
**11.3**	92/ 26.76	78/ 24.23	77/ 27.21	98/ 50.83	97/ 32.55	72/ 24.70	90/ 29.18	95/ 28.61	92/ 26.76	96/ 36.54	78/ 23.45	90/ 24.49	37/ 36.84	16/ 27.27	7/ 42.86	100/ 100	7/ 25.93
**12**	39/ 36.84	90/ 25.14	86/ 22.69	90/ 19.83	39/ 24.07	22/ 42.86	90/ 22.75	91/ 23.99	39/ 36.84	72/ 23.10	84/ 23.19	85/ 22.75	32/ 30.95	28/ 23.08	2/ 54.55	7/ 25.93	100/ 100

Table S5. Distribution of complete prophages among major clonal complexes (CC) in dataset 2.

CLONAL COMPLEX (total number of genomes)	NUMBER OF PROPHAGES
1 (260)	136
7 (7)	5
12 (38)	31
17 (90)	44
19 (29)	23
22 (3)	0
23 (56)	30
26 (4)	3
67 (1)	0
103 (2)	1
130 (2)	0
260/261 (2)	1
283 (5)	5
609 (1)	1

Table S6. Distribution of complete prophages classified based on their integrase types among major sequence types (ST) in dataset 2.

PROPHAGE	SEQUENCE TYPE (total number of isolates)				
INTEGRASE	ST1 (147)	ST17 (77)	ST19 (16)	ST23 (28)	ST459 (85)
GBS*Int*1	-	6	-	-	-
GBS*Int*2.1	-	-	-	-	1
GBS*Int*2.2	-	-	1	-	52
GBS*Int*3	20	-	7	6	13
GBS*Int*4	-	9	2	-	1
GBS*Int*6.1	-	-	1	-	-
GBS*Int*6.2	1	-	1	1	-
GBS*Int*8	1	4	-	-	-
GBS*Int*9.1	-	1	-	-	-
GBS*Int*9.2	-	7	-	-	2
GBS*Int*10	-	2	-	-	-
GBS*Int*11.1	-	1	2	1	13
GBS*Int*11.2	8	4	1	3	10
GBS*Int*12	-	-	2	-	-

Table S7. Distribution of complete prophages among group B *Streptococcus* genomes from different continents in dataset 2.

CONTINENT (total number of genomes)	NUMBER OF PROPHAGES
Africa (1)	1
Asia (10)	5
Europe (117)	68
North America (351)	195
Oceania (18)	9
South America (2)	0
Unknown (4)	2

2.1 Figures

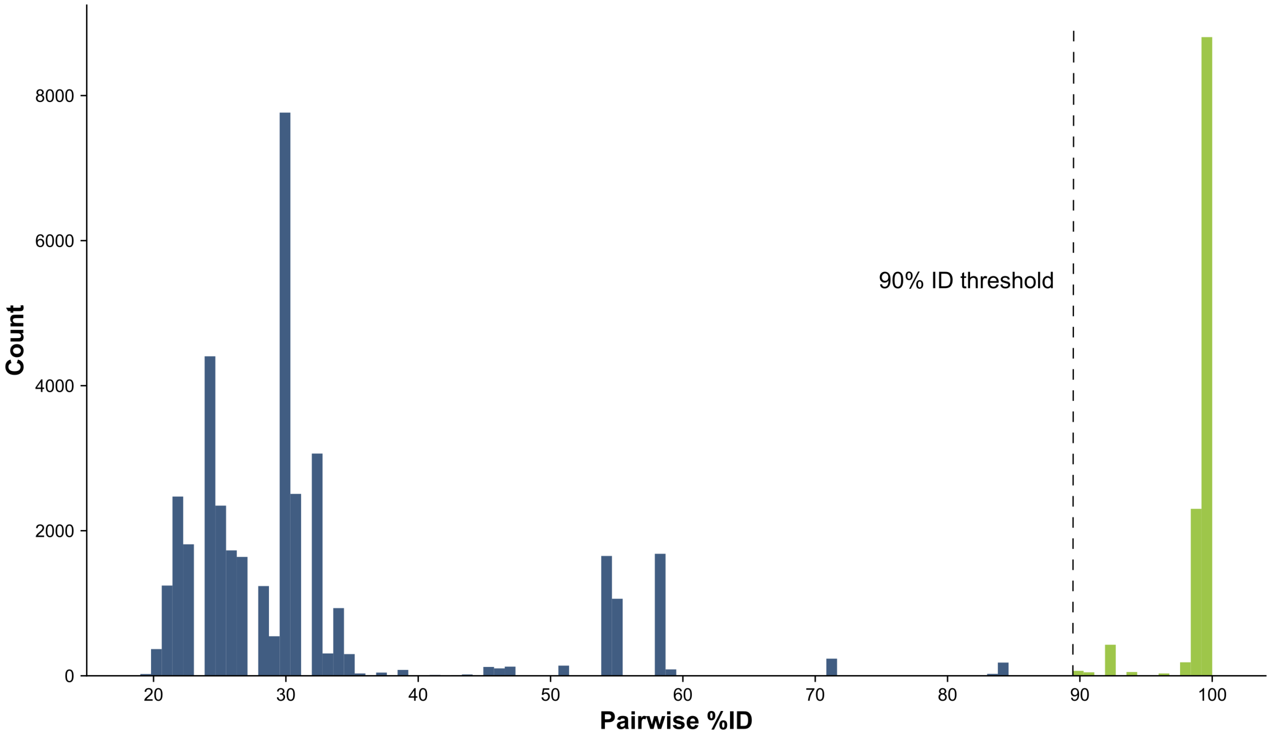

Figure S1. Histogram plot showing the distribution of the BLASTp percentage of identity (%ID) scores between all pairs of group B *Streptococcus* prophage integrase amino acid sequences identified in this study. Green bars represent %ID of matching integrase type pairs, blue bars show %ID of unmatched pairs. A minimum threshold of 90%ID BLASTp score (dashed line) was adopted to consider two integrase protein sequences as the same.

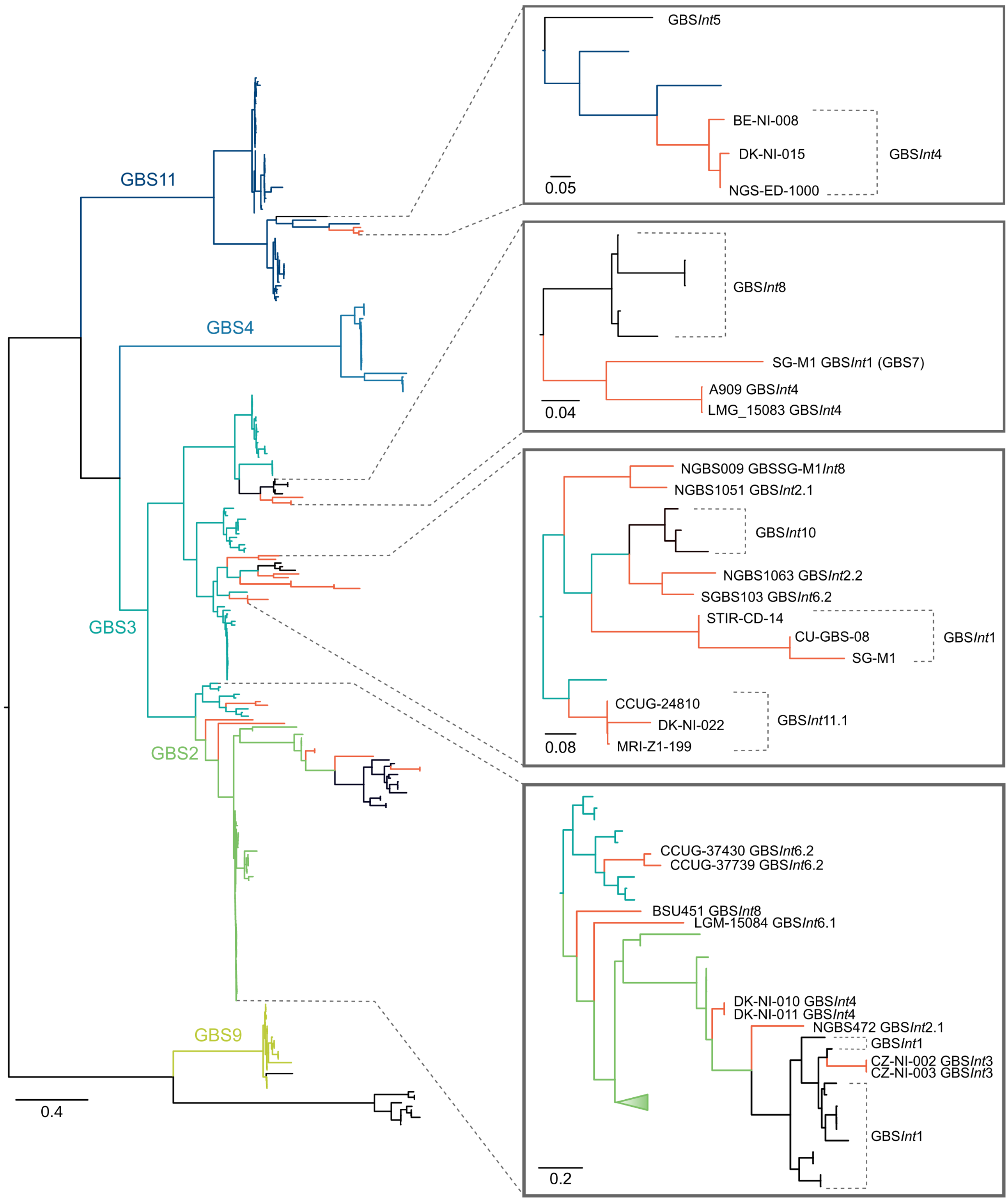

Figure S2. Approximate-maximum-likelihood phylogenetic tree of 266 complete prophages from group B *Streptococcus* (GBS) identified in dataset 2 and 22 prophages from van der Mee-Marquet et al. (2018). Phage clusters that are concordant with a particular insertion site and its integrase type/subtypes have been indicated (GBS2: green; GBS3: turquoise; GBS4: blue; GBS9: yellow; GBS11: navy). Red branches correspond to prophages that cluster within a group of phages with a different insertion site. Magnifications of such exceptions are shown and the integrase type has been indicated. Black branches correspond to minor clusters (GBS1, GBS5, GBS8, GBS10, GBS12) that are embedded in larger ones.

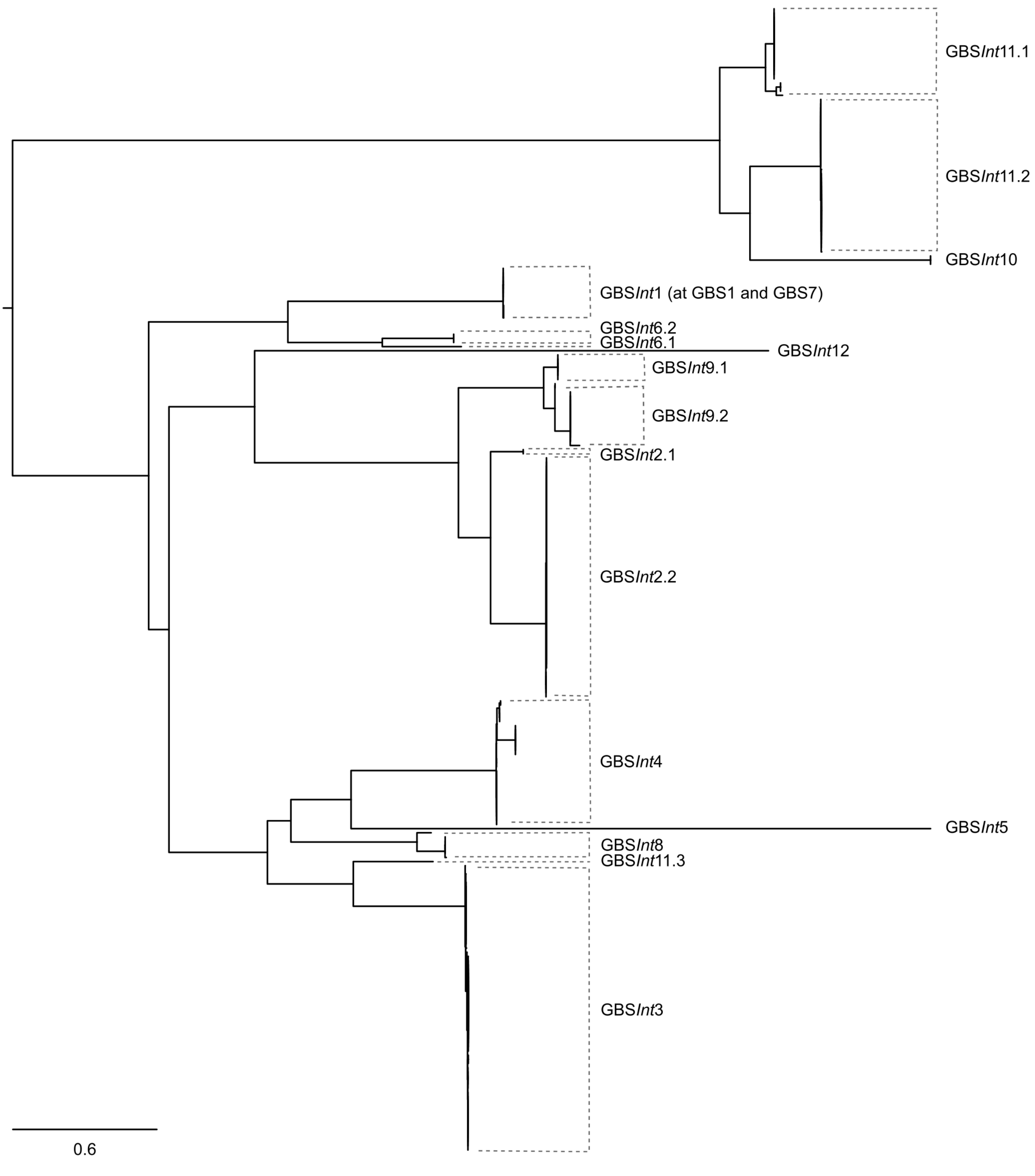

Figure S3. Approximately-maximum-likelihood phylogenetic tree of 266 phage integrase protein sequences identified in group B *Streptococcus* (GBS) in dataset 2. Integrases of the same type largely clustered within their assigned group, with the exception of GBS*Int*11.3 which clustered separately from GBS*Int*11.1 and GBS*Int*11.2.

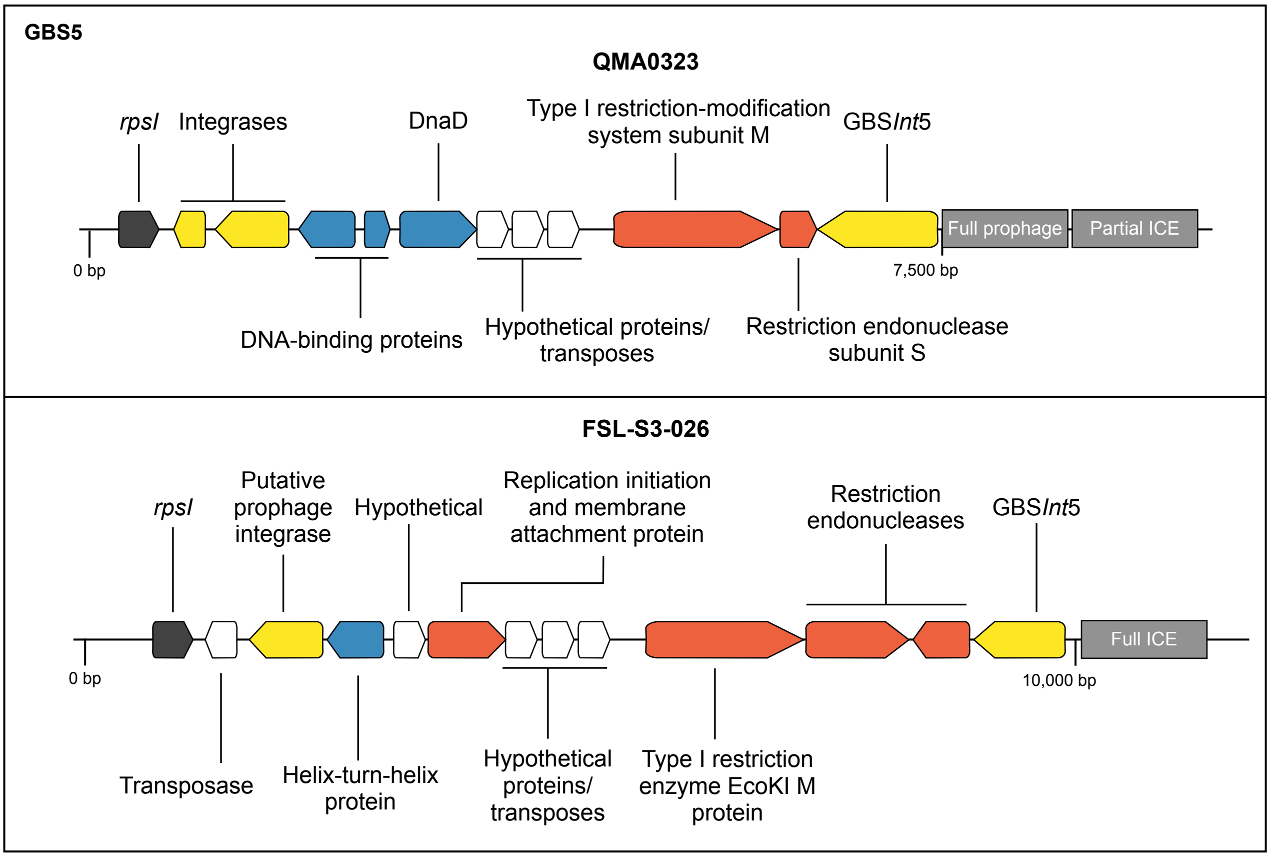

Figure S4. GBS5 insertion site as observed in group B *Streptococcus* (GBS) genome QMA0323, where GBS*Int*5 is followed by a full prophage and by part of an integrative conjugative element (ICE), and in genome FSL_S3-026, where the integrase was found as a singleton within a larger ICE. The gene *rpsI* is the closest upstream chromosomal gene to GBS*Int*5. The partial ICE after the prophage in QMA0323 showed similarity with part of the ICE in FSL_S3-026 (∼9,000bp, grey boxes at the right hand side). Genes are color-coded based on function (black: chromosomal genes; yellow: site-specific integrase; dark blue: transcriptional regulators; white: hypothetical; red: other genes).

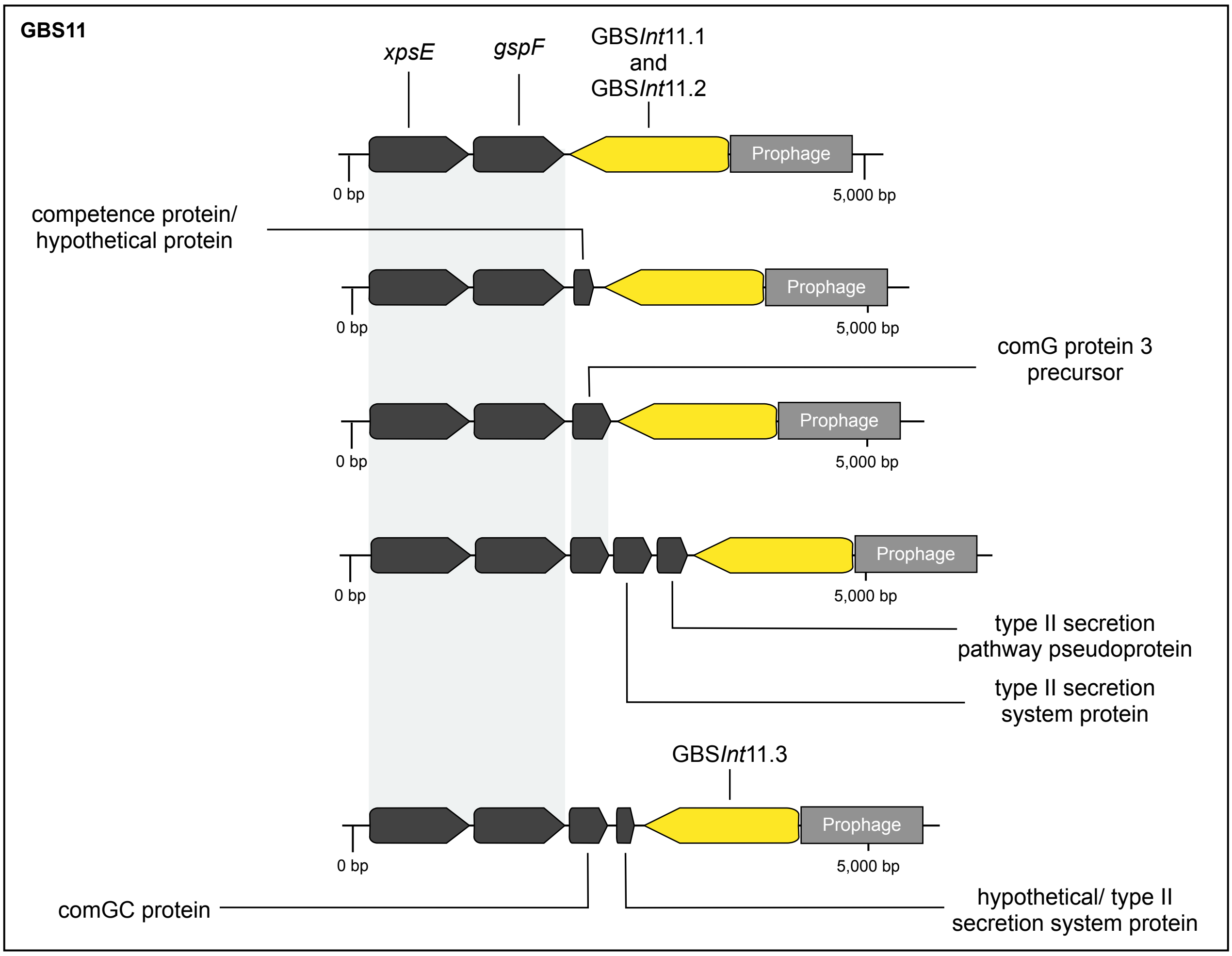

Figure S5. GBS11 insertion site variations in group B *Streptococcus* (GBS): *xpsE* and *gspF* genes are always present, and may be followed directly by GBS*Int*11.1 or GBS*Int*11.2 and the rest of the prophage. In other cases, additional small genes for competence and secretion systems were inserted between *gpsF* and the prophage, regardless of integrase type, with GBS*Int*11.3 also found in this configuration. Genes are color-coded based on function (black: chromosomal genes; yellow: site-specific integrase).

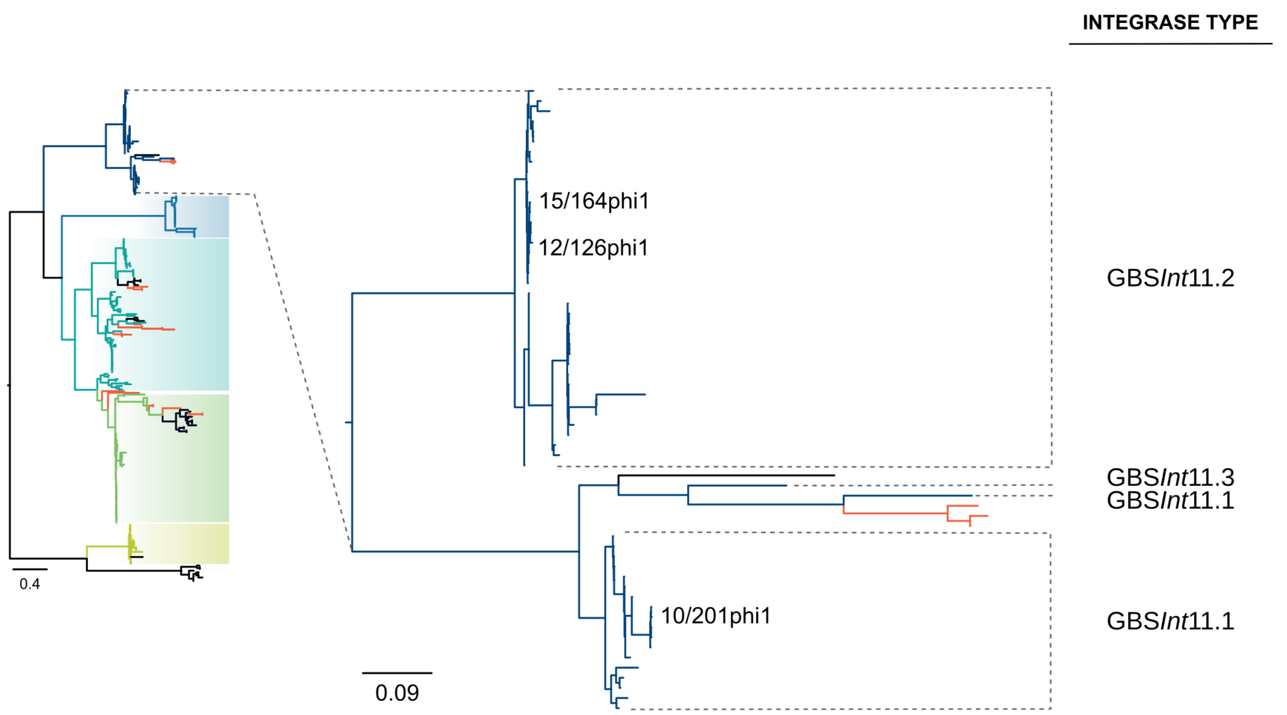

Figure S6. Magnification of the approximately-maximum-likelihood phylogenetic tree cluster of prophages with insertion site GBS11 (integrase types GBS*Int*11.1, GBS*Int*11.2 and one example of GBS*Int*11.3). Prophages F1 (12/126phi1 and 15/164 phi1 in GBS*Int*11.2) and F2 (10/201phi1 in GBS*Int*11.1) from van der Mee-Marquet et al. (2018) have been indicated (blue branches: phages with either GBS*Int*11.1, GBS*Int*11.2 or GBS*Int*11.3; black branch: prophage GBS*Int*5; red branches: prophages with GBS*Int*4).

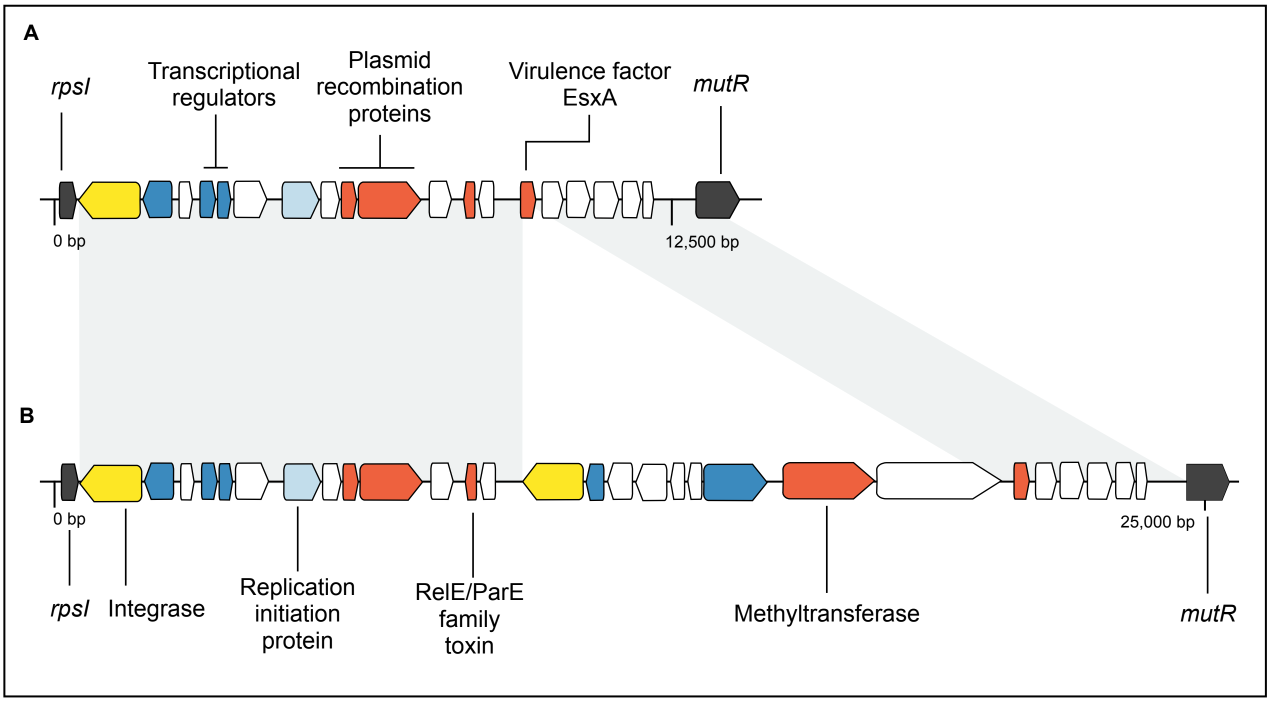

Figure S7. Example of a putative phage-inducible chromosomal island (PICI) mobile element (A) detected in the integration site *rpsI* (example genome: CU_GBS_98). This gene is described as a hotspot for recombination of mobile genetic elements (MGE): integrative conjugative elements (ICE) (Brochet al., 2008) and prophages (this work). The presence in this site of multiple site-specific integrase genes (B) is indicative of successive integration events (example genome: 09mas018883). Because of these multiple insertion events it was not possible to classify these elements with certainty, as they could have been fragments of other elements. Genes are color-coded based on function (black: chromosomal genes; yellow: site-specific integrase; dark blue: transcriptional regulators; light blue: replication genes; white: hypothetical; red: other genes).

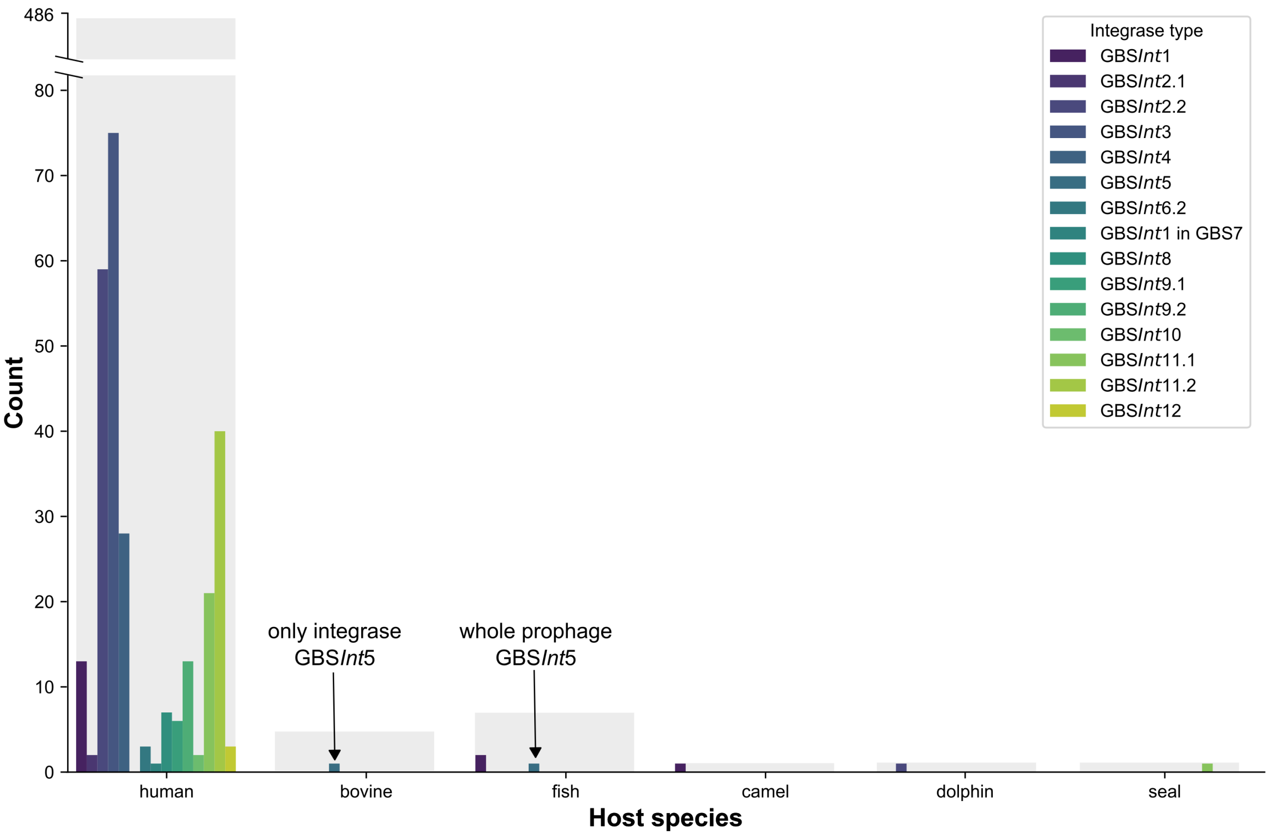

Figure S8. Distribution of complete prophages classified based on their integrase types (GBS*Int*1 to GBS*Int*12) in a publicly available Group B *Streptococcus* (GBS) dataset of 503 sequences comprising genomes from seven different host species and originating from different countries. Colored bars refer to complete prophages, with the exception of the bovine blue bar, which refers to integrase GBS*Int*5, as a singleton, i.e. not associated with a full prophage. Gray bars show the total number of isolates per host species. No prophage or integrase was detected in the single canine GBS genome included in the study.
